# Immunological heterogeneity in rheumatoid arthritis: challenges in early-stage stratification, non-response to targeted therapy, and the restoration of immune tolerance

**DOI:** 10.3389/fimmu.2026.1894263

**Published:** 2026-07-20

**Authors:** Yanmei Li, Kairui Zhu, Yushan Ding, Dongyang Wu, Yulong Mu, Xiaoke Liu, Xuelin Liu, Xinran Yu, Yuhan Yan, Yunxiao Sun, Youjie Li

**Affiliations:** 1 Department of Rheumatology and Immunology, Yantaishan Hospital Affiliated to Shandong Medical and Pharmaceutical University, Yantai, Shandong, China; 2 Department of Biochemistry and Molecular Biology, Shandong Medical and Pharmaceutical University, Yantai, Shandong, China; 3 Department of Pediatrics, Yantaishan Hospital Affiliated to Shandong Medical and Pharmaceutical University, Yantai, Shandong, China

**Keywords:** autoantibody diversification, gut-joint axis, immunological heterogeneity, molecular subtyping, precision stratification, rheumatoid arthritis, synovial pathotype, treatment non-response

## Abstract

Rheumatoid arthritis (RA) is a systemic autoimmune disease characterized by chronic erosive synovitis, progressive bone destruction, and marked inter-patient heterogeneity in disease course and treatment response. This review critically examines the mechanisms underlying RA immunological heterogeneity, including genetic susceptibility, epigenetic regulation, autoantibody diversification, synovial pathotypes, and gut-joint axis-related immune-metabolic interactions. We further discuss how peripheral blood multi-omics, synovial molecular pathology, and biopsy-driven clinical trial evidence may inform early stratification and prediction of primary non-response. Rather than proposing a deterministic precision-medicine model, this review emphasizes an evidence-weighted framework in which molecular and tissue-level biomarkers are integrated with clinical phenotype and longitudinal treatment response. Emerging approaches, including pharmacomicrobiomics, local drug delivery, nanomedicine, and cell-based therapies, are evaluated as investigational strategies that require further validation. Overall, this review highlights how a more critical understanding of RA heterogeneity may improve patient stratification and support more rational therapeutic selection.

## 1 Introduction

Rheumatoid arthritis (RA) is a chronic systemic autoimmune disease characterized by persistent synovitis, pannus formation, and progressive cartilage and bone damage (1). If inadequately controlled, sustained immune-mediated inflammation may contribute to joint deformity, functional disability, and substantial socioeconomic burden (2). Despite advances in diagnosis and treatment, RA remains difficult to manage because patients differ markedly in age at onset, autoantibody status, inflammatory burden, synovial tissue organization, structural progression, and response to targeted therapy (3, 4).

RA heterogeneity arises from the interaction of multiple biological layers rather than from a single pathogenic pathway. Genetic susceptibility, particularly within human leukocyte antigen (HLA) and non-HLA immune-regulatory loci, shapes antigen presentation and baseline autoimmune risk (5, 6). Epigenetic regulation, post-transcriptional networks, and immune-metabolic remodeling further influence immune-cell activation and synovial stromal behavior (5). In parallel, autoantibody diversification, including anti-citrullinated protein antibodies (ACPAs) and broader anti-modified protein antibodies (AMPAs), provides a major axis of patient stratification (7, 8). The gut-joint axis and microbiome-associated metabolic changes have also been linked to RA-related immune dysregulation, although their causal role in human disease remains incompletely defined (9, 10).

Clinically, conventional synthetic disease-modifying antirheumatic drugs (csDMARDs), biological DMARDs (bDMARDs), and targeted synthetic DMARDs (tsDMARDs) have improved outcomes, but primary non-response and secondary loss of response remain common challenges (11, 12). Peripheral biomarkers, imaging, and synovial molecular pathology may help refine patient stratification, but current evidence does not support a simple one-marker-one-drug model (5, 13). Biopsy-driven trials such as R4RA and STRAP highlight both the promise and limitations of synovial pathotype-guided therapy (14). Unlike previous reviews that mainly summarize individual pathogenic pathways, omics biomarkers, or therapeutic classes, this review integrates these dimensions into an evidence-weighted translational framework linking RA immunological heterogeneity with early stratification, treatment non-response, and immune rebalancing strategies. Therefore, this review critically examines the mechanisms and clinical implications of RA immunological heterogeneity, with emphasis on evidence-weighted stratification, treatment non-response, and emerging but still investigational therapeutic strategies (11, 15).

## 2 Sources and mechanisms of RA immunological heterogeneity

Clinical variability in RA reflects the dynamic evolution of immunological heterogeneity across disease stages, tissue compartments, and immune-cell populations. Patients differ in age at onset, joint distribution, inflammatory burden, autoantibody profiles, rate of structural damage, and response to therapy. These differences indicate that RA is not driven by a single immune defect, but by interactions among genetic susceptibility, epigenetic regulation, post-transcriptional networks, and tissue-specific immune remodeling (16, 17). Clarifying how these layers interact is essential for understanding RA heterogeneity and for developing more reliable approaches to early stratification and treatment-response prediction.

### 2.1 Genetic architecture as a baseline layer of disease heterogeneity

Genetic background represents an important baseline layer of RA immunological heterogeneity rather than a simple inherited determinant (17). Variants within HLA and non-HLA immune-regulatory loci may influence antigen presentation, immune-cell activation thresholds, cytokine signaling, autoantibody profiles, structural damage risk, and treatment response (18). Thus, genetic susceptibility contributes not only to disease risk but also to the formation of distinct molecular and clinical phenotypes (19).

Among genetic factors, the HLA-DRB1 shared epitope (SE) remains the best-characterized risk architecture for ACPA-positive RA (20). SE alleles alter the antigen-binding groove of HLA-DR molecules and may facilitate the presentation of citrullinated peptides to autoreactive T cells, thereby supporting downstream B-cell activation and ACPAs production (21). However, HLA-associated risk is not uniform across populations; risk and protective alleles vary by ancestry, geography, and environmental background (22). Therefore, HLA status should be interpreted as a major susceptibility and stratification factor rather than as a deterministic predictor of disease course.

Non-HLA susceptibility genes further diversify RA immune phenotypes. Variants in PTPN22, STAT4, CTLA4, CD28, TRAF3IP2, and cytokine-related loci may alter T-cell regulation, co-stimulatory signaling, inflammatory amplification, and the balance between pro-inflammatory and regulatory pathways (23). These effects may bias patients toward different immune programs, such as Th17-skewed inflammation, B-cell-driven autoimmunity, or cytokine-dominant disease, but they do not act in isolation. Genetic susceptibility interacts with smoking, periodontal pathogens, mucosal citrullination, and other environmental exposures to shape the timing and site of immune initiation (9). This gene-environment interaction helps explain why patients with similar clinical diagnoses may differ in autoantibody repertoires, inflammatory pathways, bone damage, and treatment response. Nevertheless, DNA variation provides only a relatively stable risk framework; the conversion of genetic risk into persistent inflammation depends on dynamic regulatory layers, including epigenetic remodeling (24).

### 2.2 Epigenetic regulation and cellular plasticity

While genetic susceptibility provides a relatively stable risk framework, epigenetic regulation may help explain how environmental and inflammatory signals are translated into persistent changes in immune-cell and stromal-cell function (25). DNA methylation, histone modification, and metabolite-linked chromatin remodeling are dynamic and context-dependent processes that can respond to smoking, infection, mucosal barrier disruption, metabolic stress, and local synovial inflammation (26).

Aberrant DNA methylation has been reported in both circulating immune cells and synovial resident cells in RA (27). Hypomethylation of selected inflammatory genes may increase cytokine or chemokine expression, whereas hypermethylation of regulatory loci may weaken anti-inflammatory programs (28). For example, altered methylation of genes related to STAT3 and transforming growth factor-β (TGF-β) signaling has been associated with imbalance between T helper 17 (Th17) cells and regulatory T cells (Tregs). In the synovium, epigenetic changes in macrophages, fibroblast-like synoviocytes (FLS), and lymphocytes may contribute to inflammatory persistence, stromal activation, and tissue invasion (29). However, these associations should be interpreted cautiously, because many findings are cell-type-, disease-stage-, and platform-dependent (30).

Histone modifications provide another layer of regulation by altering chromatin accessibility and transcriptional output. Changes in histone acetylation and methylation may facilitate inflammatory transcriptional programs, increase cytokine and matrix metalloproteinases (MMPs) expression, and influence FLS activation (31). More recently, metabolite-linked epigenetic mechanisms have drawn attention (32). Glycolysis-associated lactate accumulation may induce histone lactylation marks, such as H3K18la and H3K9la, which have been linked to FLS proliferation, ferroptosis resistance, and Th17-skewed inflammation in experimental settings (33). These findings suggest a possible connection between metabolic remodeling and epigenetic regulation, although their clinical significance in human RA remains under investigation.

From a translational perspective, epigenetic profiles may provide candidate biomarkers for disease activity, structural progression, and treatment-response prediction (34). Because epigenetic states are potentially reversible, they also represent attractive therapeutic targets (31). Nevertheless, clinical application remains limited by tissue heterogeneity, assay variability, cost, and the lack of large prospective validation studies. At present, epigenetic profiling should therefore be viewed as a promising but still exploratory component of RA stratification rather than a routine clinical decision tool.

### 2.3 Post-transcriptional regulation by ncRNAs and epitranscriptomic modifications

Beyond genetic and epigenetic regulation, post-transcriptional mechanisms further refine RA immunological heterogeneity (35). Non-coding RNAs (ncRNAs) and epitranscriptomic modifications can influence transcript stability, translation, immune-cell activation, and stromal-cell behavior (36). These regulatory layers are highly cell-type- and disease-stage-dependent and may help explain why similar upstream stimuli produce different inflammatory and tissue-destructive phenotypes across patients (37).

Among epitranscriptomic modifications, N6-methyladenosine (m6A) is the most widely studied in RA (38). The balance among m6A “writers,” “erasers,” and “readers” may alter messenger RNA (mRNA) stability and translation in macrophages, FLS, and other synovial cell populations (39). Emerging evidence suggests that altered m6A regulation can affect transcripts involved in glycolysis, inflammatory signaling, and hypoxia responses, thereby linking RNA modification to metabolic remodeling and FLS activation (40). However, most evidence remains mechanistic or observational, and the clinical utility of m6A-related markers in RA stratification still requires validation.

ncRNAs provide another post-transcriptional layer (41). MicroRNAs (miRNAs), long non-coding RNAs (lncRNAs), and circular RNAs (circRNAs) can regulate inflammatory pathways, cytokine production, angiogenesis, and matrix-degrading programs through direct transcript targeting or competing endogenous RNA (ceRNA) networks (41). For example, dysregulated circRNAs or lncRNAs may sponge miRNAs that normally restrain MMPs expression, vascular endothelial growth factor signaling, or fibroblast activation (42). In addition, ncRNAs may interact with epitranscriptomic machinery by influencing m6A-modifying enzymes, while m6A can affect ncRNA processing and stability (43). This crosstalk suggests that post-transcriptional regulation may contribute to heterogeneous synovial cell states, although pathway-specific causality in human RA remains incompletely established.

From a translational perspective, circulating or exosome-associated ncRNAs are attractive candidate biomarkers because of their relative stability and accessibility in peripheral blood (44). They may partially reflect synovial inflammation, treatment response, or structural damage risk, but they should not be assumed to faithfully represent the *in situ* synovial microenvironment (45). At present, ncRNA and m6A-related markers are best viewed as exploratory tools for molecular stratification rather than routine clinical tests. Direct therapeutic targeting of ncRNAs or epitranscriptomic enzymes also remains challenging because of off-target effects, delivery barriers, tissue specificity, and the physiological roles of these pathways. Therefore, post-transcriptional regulation provides an important mechanistic layer of RA heterogeneity, but its clinical translation will require standardized assays, longitudinal validation, and safer tissue-targeted delivery strategies.

### 2.4 Spatiotemporal evolution of RA immunological heterogeneity

RA immunological heterogeneity is not a static feature captured by a single biomarker or a single sampling time point. Instead, it evolves across disease stages, tissue compartments, and treatment timelines (46). Genetic susceptibility, epigenetic regulation, and post-transcriptional networks provide important molecular layers, but the clinical phenotype of an individual patient is shaped by how these layers are remodeled over time and across anatomical sites. This spatiotemporal nature helps explain why peripheral blood, mucosal tissue, and synovial tissue may provide complementary but non-equivalent information.

Along the temporal axis, RA may begin with preclinical mucosal immune perturbation, environmental exposure, early autoantibody development, and low-grade systemic inflammation before clinically apparent arthritis emerges (47). During early arthritis, innate immune-cell recruitment, antigen presentation, lymphocyte activation, and cytokine amplification generate inter-patient differences in autoantibody repertoires and inflammatory intensity (40). In established RA, synovial tissue becomes a dominant effector site, where FLS activation, angiogenesis, osteoclastogenesis, and extracellular matrix remodeling contribute to structural damage (48). With chronic disease and repeated treatment exposure, inflammatory networks may be reshaped by tissue fibrosis, stromal persistence, compensatory signaling, and treatment-associated immune remodeling (49). Therefore, biomarkers obtained at one disease stage may not reliably predict later tissue behavior or therapeutic response.

Along the spatial axis, immune activity differs across peripheral blood, mucosal sites, synovial tissue, and even between joints. Peripheral blood reflects systemic immune activity but may not accurately represent local synovial invasion. Mucosal tissues may capture early events related to immune tolerance disruption, whereas synovial tissue reflects local effector mechanisms such as persistent inflammation, stromal activation, and bone destruction. Synovial studies have identified major pathotypes, including lympho-myeloid, diffuse-myeloid, and pauci-immune/fibroid patterns (50). However, their therapeutic implications should be interpreted cautiously and are discussed in detail in Section 5.2.

Treatment itself can further remodel RA immune heterogeneity. Blocking dominant cytokine pathways, such as TNF-α or IL-6 signaling, may reduce inflammation in some patients but also reveal or select alternative inflammatory programs, including JAK/STAT- or interferon-associated pathways (49). In others, tissue fibrosis, stromal activation, or anti-drug antibodies (ADAs) may contribute to altered treatment sensitivity (51). These observations indicate that treatment response is dynamic rather than fixed at baseline.

**Figure 1:**
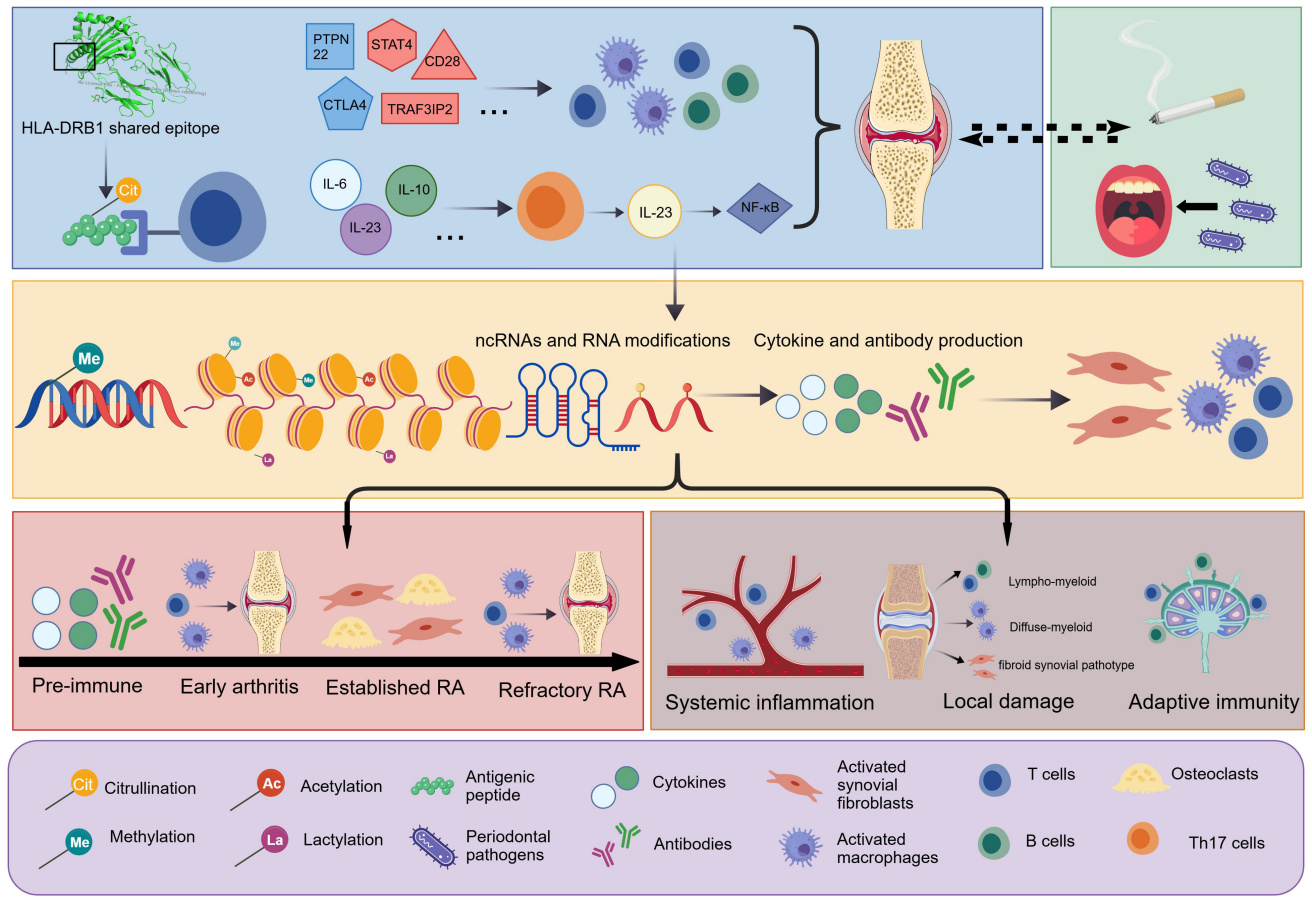
Multilayered mechanisms and clinical evolution of RA immunological heterogeneity. Genetic susceptibility, environmental exposures, mucosal immune disturbance, epigenetic regulation, post-transcriptional networks, cytokine-network amplification, and autoantibody diversification jointly shape RA immunological heterogeneity. These mechanisms evolve across preclinical autoimmunity, early arthritis, established RA, and refractory RA, contributing to systemic inflammation, adaptive immune dysregulation, structural damage, distinct synovial pathotypes, and tissue-destructive microenvironments.

The clinical implication is that RA stratification should integrate disease stage, tissue source, and longitudinal change rather than rely on a single static biomarker. Static genetic profiling may help define baseline susceptibility, whereas epigenetic, transcriptomic, and post-transcriptional markers may provide more dynamic information but remain limited by assay variability, cost, tissue heterogeneity, and insufficient prospective validation (**Table 1**). Thus, spatiotemporal heterogeneity provides a rationale for multidimensional and longitudinal assessment, while also cautioning against overinterpreting any single molecular layer as a potential guide for precision therapy (**Figure 1**).

**Table 1:** Mechanistic sources of RA immunological heterogeneity and their translational implications.

Mechanistic layer	Core elements	Contribution to RA immunological heterogeneity	Stratification or clinical value	Evidence status and translational limitation	References
Genetic architecture	HLA-DRB1 SE; non-HLA immune-regulatory loci such as PTPN22, STAT4, CTLA4, CD28, TRAF3IP2, and cytokine-related genes	Establishes baseline autoimmune susceptibility and influences antigen presentation, immune-cell activation thresholds, autoantibody profiles, inflammatory pathway bias, structural damage risk, and treatment-response tendency	Supports baseline risk assessment and helps contextualize ACPA-positive RA, autoantibody breadth, and pathway-dependent treatment responses	Strong genetic association evidence, but limited predictive value when used alone; effects vary by ancestry, geography, and environmental background	(52, 53)
Gene-environment interaction and mucosal immune triggering	Smoking, periodontal pathogens, mucosal citrullination, environmental exposures, and early barrier disturbance	Converts genetic susceptibility into immune activation by increasing modified antigen burden, disrupting mucosal immune regulation, and promoting early systemic autoimmunity	May help identify high-risk individuals in preclinical or early RA, particularly in ACPA-positive RA	Biologically plausible and epidemiologically supported, but exposure quantification and causal direction remain difficult to standardize	(54–56)
DNA methylation	Hypomethylation of inflammatory genes; hypermethylation of regulatory loci	Alters cytokine and chemokine expression, Th17/Treg balance, immune-cell activation, stromal activation, and persistence of local inflammation	Candidate biomarker layer for disease activity, structural progression, and treatment-response prediction	Promising but exploratory; findings are cell-type-, disease-stage-, and platform-dependent, with limited prospective validation	(57–59)
Histone modification and metabolism-linked chromatin remodeling	Histone acetylation, histone methylation, and lactate-associated histone lactylation	Regulates inflammatory transcriptional programs, MMP expression, FLS activation, ferroptosis resistance, and Th17-skewed inflammation	Provides a mechanistic bridge between immune-metabolic remodeling and persistent synovial inflammation	Mechanistic evidence is growing, but clinical significance in human RA and therapeutic specificity remain insufficiently validated	(60, 61)
Post-transcriptional regulation	m6A modification; miRNAs, lncRNAs, circRNAs, and ceRNA networks	Modulates mRNA stability, translation, cytokine production, angiogenesis, matrix-degrading programs, FLS activation, and immune-cell function	Circulating or extracellular vesicle-associated RNA markers may support non-invasive molecular stratification and longitudinal monitoring	Mainly mechanistic or observational; tissue specificity, assay standardization, delivery barriers, and off-target effects limit translation	(62, 63)
Tissue-compartment heterogeneity	Peripheral blood, mucosal tissues, synovial tissue, and inter-joint variation	Generates non-equivalent immune information across compartments; systemic biomarkers may not fully reflect local synovial pathology	Supports integrated interpretation of peripheral biomarkers, mucosal signals, imaging, and synovial molecular pathology	Conceptually strong, but tissue sampling is clinically challenging and synovial accessibility remains limited	(64, 65)
Disease-stage evolution	Preclinical autoimmunity, early arthritis, established RA, and refractory RA	Dominant pathogenic mechanisms may shift from mucosal immune perturbation and autoantibody development to synovial stromal remodeling and treatment-resistant tissue pathology	Provides rationale for stage-specific stratification and longitudinal biomarker monitoring	Biomarkers measured at one disease stage may not reliably predict later tissue behavior or therapeutic response	(66, 67)
Treatment-induced immune remodeling	Cytokine blockade, JAK inhibition, ADAs, compensatory signaling, pathway switching, and stromal persistence	Therapeutic pressure may suppress one inflammatory pathway while revealing or selecting alternative immune, stromal, or pharmacokinetic mechanisms of persistence	Helps explain primary non-response, secondary loss of response, and the need for dynamic reassessment during treatment	Mechanisms of therapeutic escape are heterogeneous and require integration of clinical activity, drug exposure, ADAs, imaging, and molecular data	(68, 69)
Integrated dynamic heterogeneity	Interaction among genetic, epigenetic, post-transcriptional, spatial, temporal, and treatment-related layers	Explains why patients with the same clinical diagnosis may differ in autoantibody status, synovial pathotype, structural progression, and treatment response	Supports transition from static diagnosis toward mechanism-informed stratification and dynamic monitoring	Precision models remain limited by assay variability, cost, interpretability, and insufficient prospective validation	(70, 71)

## 3 Imbalances in the pathogenic immune network and the formation of a tissue-destructive microenvironment

Genetic, epigenetic, and post-transcriptional regulation shape the upstream basis of RA heterogeneity (17), but disease progression depends on how these molecular layers are translated into dysfunctional innate and adaptive immune responses. RA is therefore not sustained by a single inflammatory mediator, but by interconnected immune-cell subsets, cytokine networks, stromal-cell interactions, and intracellular signaling pathways (72). These networks can initiate local inflammation, amplify adaptive immune dysregulation, promote autoantibody-associated immune activation, and reshape resident synovial cells (73).

As RA progresses, the synovium develops a tissue-destructive microenvironment characterized by inflammatory-cell infiltration, FLS activation, angiogenesis, osteoclastogenesis, and extracellular matrix remodeling (74). This transition helps explain how initially reversible inflammation may evolve into persistent synovitis and structural damage. Understanding how pathogenic immune networks are initiated, amplified, and spatially organized is therefore essential for explaining inter-patient differences in clinical phenotype, tissue destruction, treatment response, and therapeutic resistance.

### 3.1 Innate immune activation and inflammatory initiation

Innate immune activation is an early component of RA inflammation. Synovial tissue injury, mucosal antigens, immune complexes, and DAMPs may activate macrophages, dendritic cells (DCs), neutrophils, and mast cells (75). In genetically and epigenetically susceptible individuals, these cells may show lower activation thresholds and prolonged inflammatory output, thereby creating a local environment that supports subsequent adaptive immune activation (76).

Synovial macrophages represent a central but heterogeneous myeloid compartment in RA. Their function should no longer be interpreted through a simple M1/M2 polarization model. Single-cell transcriptomic, mass cytometry, and spatial profiling studies have shown that RA synovium contains multiple macrophage states with distinct origins, tissue locations, transcriptional programs, and functional outputs (77). These include monocyte-derived inflammatory macrophage states enriched for IL1B, CXCL chemokines, HBEGF, SPP1, or SLAMF7-related programs, as well as tissue-resident or remission-associated macrophage subsets characterized by markers such as MERTK and CD206 (78). Thus, synovial macrophages are better viewed as a dynamic cell-state continuum shaped by cytokines, immune complexes, metabolic stress, stromal interactions, and treatment pressure.

Functionally, inflammatory macrophage states may produce TNF-α, IL-6, IL-1β, chemokines, and tissue-remodeling mediators, thereby recruiting leukocytes, activating endothelial cells, and amplifying myeloid–stromal crosstalk (79). Conversely, resident lining macrophages and MERTK-positive synovial macrophages have been implicated in barrier maintenance, efferocytosis, inflammation resolution, and remission stability (80). This duality cautions against treating all synovial macrophages as uniformly pathogenic. RA progression and treatment response may depend on the relative abundance, spatial organization, and functional coupling of discrete macrophage states within a given synovial pathotype (81).

DCs provide an important link between innate immune sensing and adaptive immune activation (82). Under persistent inflammatory stimulation and exposure to modified autoantigens, including citrullinated antigens, DCs may increase antigen presentation, co-stimulatory signaling, and cytokine production (83). These changes can promote CD4+ T-cell activation and support the expansion of Th1, Th17, and Tfh cell responses (83). Therefore, DCs may contribute to the transition from local innate inflammation to broader adaptive immune dysregulation, although their effects are likely context-dependent and shaped by tissue niche and disease stage.

Neutrophils also amplify synovial inflammation. After recruitment into the joint cavity, neutrophils can release ROS, antimicrobial peptides, and MMPs, thereby contributing to tissue injury and inflammatory amplification (84). NETosis further exposes NET-associated modified autoantigens and DAMPs, which may activate macrophages and DCs and reinforce inflammasome-related inflammatory loops (75). In this way, neutrophils may connect tissue injury, autoantigen exposure, and ACPA-associated immune activation, particularly in seropositive disease.

Mast cells are tissue-resident innate immune cells that may participate in the early amplification of synovitis (85). Upon activation by immune complexes, IgE-dependent signals, or local inflammatory cues, mast cells can release histamine, proteases, TNF-α, and other mediators that increase vascular permeability and facilitate leukocyte recruitment (86). Their rapid response capacity suggests that mast cells may help shape an early pro-inflammatory synovial niche, although their relative contribution is likely to vary across patients and disease stages.

Overall, innate immune dysregulation in RA reflects a multicellular amplification network rather than dysfunction of a single cell type. Macrophages, DCs, neutrophils, and mast cells collectively contribute to danger-signal sensing, cytokine production, autoantigen exposure, and synovial microenvironment remodeling. This innate immune network provides a foundation for subsequent adaptive immune imbalance, autoantibody production, and progressive tissue damage, while also contributing to inter-patient differences in inflammatory phenotype and early treatment response.

### 3.2 Adaptive immune dysregulation and autoantibody-driven amplification

After innate immune activation has established a pro-inflammatory synovial environment, adaptive immune dysregulation further amplifies RA pathology (87). Aberrant antigen presentation, excessive co-stimulatory signaling, and persistent cytokine stimulation may promote the survival and expansion of autoreactive T and B cells (88). This process supports the transition from local synovial inflammation to systemic autoimmunity and contributes to chronic synovitis, autoantibody production, and progressive tissue damage.

At the T-cell level, RA is characterized by imbalance among pathogenic effector T-cell subsets and insufficient regulatory control (89, 90). Under the influence of IL-6, IL-12, IL-23, and chronic antigenic stimulation, naive CD4+ T cells may differentiate toward Th1, Th17, and Tfh cell programs, whereas Treg function may be impaired. Th17 cells can promote synovial inflammation, neutrophil recruitment, FLS activation, and osteoclastogenesis through IL-17 and related mediators (91). Tfh cells support B-cell activation, germinal-center responses, and autoantibody production through co-stimulatory signals and cytokines such as IL-21 (92, 93). The relative dominance of Th17-driven inflammation, Tfh–B-cell interaction, or broader JAK/STAT-dependent signaling may differ among patients, providing a mechanistic basis for variable responses to co-stimulation blockade, B-cell depletion, cytokine inhibition, or JAK inhibitors (13).

B-cell dysregulation represents another major component of adaptive immune amplification. Autoreactive B cells and plasma cells can produce ACPAs, RF, and other autoantibodies that reflect disrupted humoral tolerance and may also contribute to pathogenic immune activation (7). Autoantibodies can form immune complexes (ICs) with modified self-antigens, deposit in synovial tissue, activate complement, and recruit macrophages and neutrophils through C3a- and C5a-mediated inflammatory pathways (93, 94). In addition, ACPAs may promote macrophage inflammasome activation, synovial-cell stimulation, osteoclast differentiation, and bone-resorptive activity in a context-dependent manner (95). Thus, autoantibodies should be viewed not only as diagnostic markers but also as potential effectors linking systemic autoimmunity with local tissue injury.

Adaptive immune responses can also become organized within the synovium. In a subset of patients, T cells, B cells, and antigen-presenting cells aggregate into tertiary lymphoid structures (TLS), which may support local antigen presentation, autoreactive lymphocyte selection, and *in situ* autoantibody production (96). This tissue-localized immune organization is particularly relevant to lympho-myeloid synovial pathotypes, although its treatment implications require cautious interpretation and are discussed further in Section 5.2.

**Figure 2:**
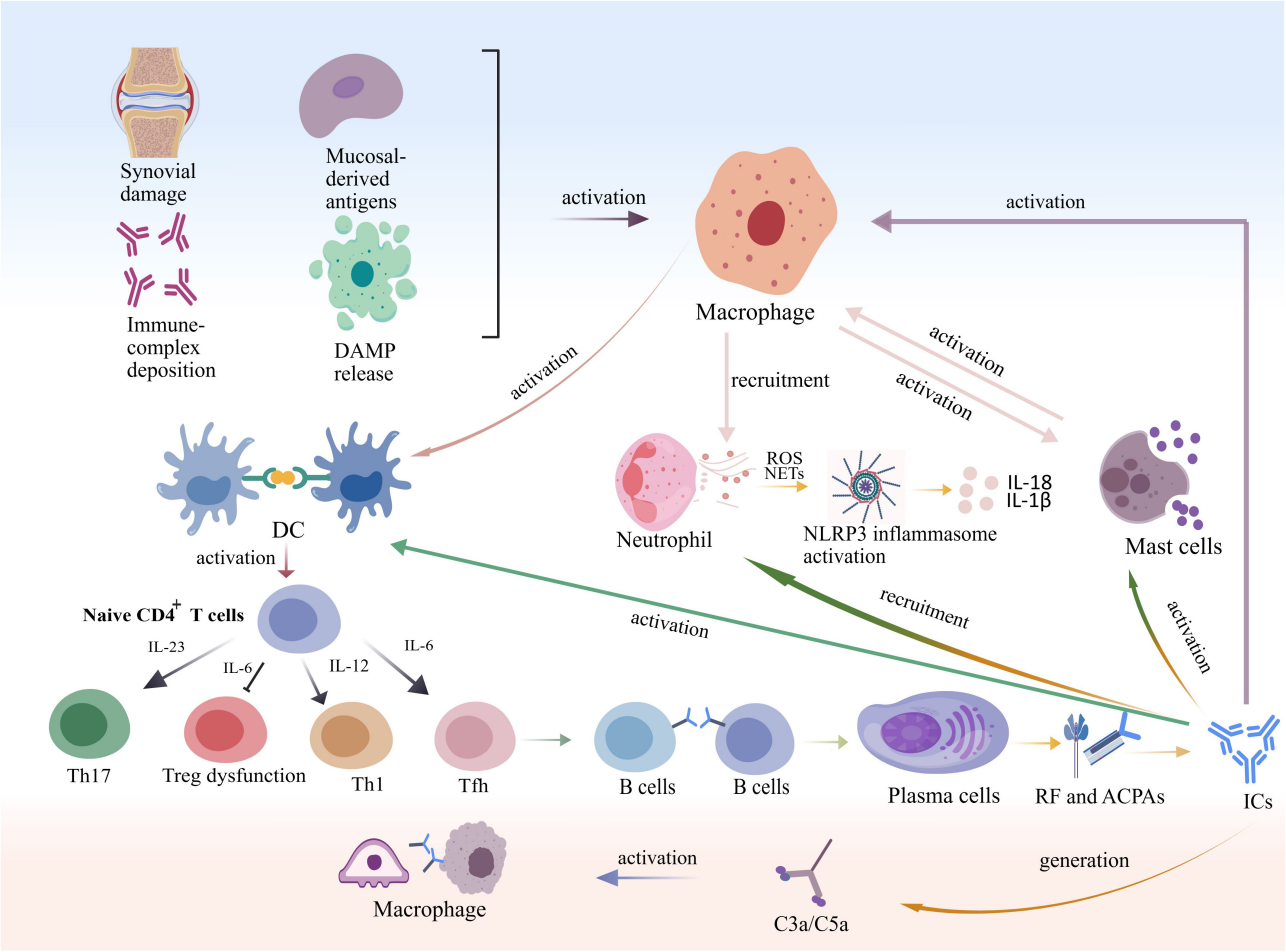
Innate immune activation, adaptive immune dysregulation, and autoantibody-driven amplification in RA. Synovial damage, mucosal antigens, DAMPs, and immune-complex deposition may activate macrophages, DCs, neutrophils, and mast cells, thereby initiating inflammatory amplification. DC-mediated antigen presentation and cytokine release promote naïve CD4+ T-cell differentiation toward Th1, Th17, and Tfh cell programs, while insufficient Treg-mediated regulation further supports adaptive immune dysregulation. Activated B cells and plasma cells produce RF and ACPAs, which form ICs and activate C3a- and C5a-mediated inflammatory pathways. These processes reinforce macrophage activation, neutrophil recruitment, NETosis, NLRP3 inflammasome activation, and IL-1β/IL-18 release, thereby creating self-sustaining inflammatory loops in RA.

Overall, adaptive immune dysregulation provides a major amplification layer in RA by linking T-cell imbalance, B-cell activation, IC formation, and local synovial immune organization. Inter-patient differences in the dominance of Th17-, Tfh–B-cell-, or IC-driven mechanisms may contribute to heterogeneity in clinical phenotype, structural progression, and treatment response (**Figure 2**).

### 3.3 ACPA and AMPA heterogeneity as a major axis of immune stratification

Although ACPA positivity is widely used in RA diagnosis and prognosis, the ACPA response should not be treated as a single serological entity (97). ACPA-positive patients may differ in antigenic breadth, fine specificity, isotype distribution, affinity maturation, tissue origin, glycosylation pattern, and downstream effector function (98). This is clinically important because two patients who are both anti-CCP positive may have different autoreactive B-cell repertoires, mucosal triggers, bone-destructive potential, and treatment trajectories (97). Therefore, ACPAs are better understood as a multidimensional immunological phenotype rather than a simple classification marker.

At the level of fine specificity, ACPAs recognize a broad but non-random spectrum of citrullinated antigens, including citrullinated vimentin, fibrinogen, type II collagen, α-enolase, histones, and filaggrin-related epitopes (99). Citrullinome studies have further identified citrullinated proteins in RA synovial fluid, such as apolipoprotein E, myeloid nuclear differentiation antigen, and β-actin, indicating that local joint biochemistry may shape the ACPA repertoire (10, 64). Among these specificities, anti-CEP-1 reactivity is particularly informative because it has been linked to smoking, HLA-DRB1 SE alleles, and PTPN22 risk variants. By contrast, reactivity against citrullinated fibrinogen or vimentin may more closely reflect intra-articular antigen availability and tissue-destructive synovitis (100). Thus, ACPA fine specificity may provide a more mechanistically informative stratification layer than the crude seropositive/seronegative distinction.

The temporal evolution of ACPA reactivity further supports its role in RA heterogeneity. During the preclinical phase, ACPA responses may begin with a limited number of specificities and later expand to multiple citrullinated epitopes, a process often described as epitope spreading (64). Longitudinal studies before RA onset suggest that individuals who later develop clinical arthritis may accumulate broader ACPA reactivity together with rising inflammatory mediators (101). However, this should be interpreted probabilistically rather than deterministically. Not all fine specificities show consistent prognostic value after early RA is established, and cohort-level associations with disease activity, erosion, or treatment response remain variable.

Another important layer is antibody glycosylation. At the Fc level, ACPA-IgG may acquire pro-inflammatory glycosylation features before clinically apparent arthritis, including reduced galactosylation and sialylation (102, 103). These changes may enhance Fcγ receptor engagement, complement activation, and macrophage cytokine release. At the Fab level, ACPAs frequently contain variable-domain N-linked glycans, which may alter antibody charge, antigen binding, and autoreactive B-cell selection (104). Therefore, ACPA pathogenicity depends not only on antigen specificity, but also on antibody structure, glycosylation, isotype, RF co-reactivity, and local inflammatory context.

AMPAs further extend this heterogeneity. In addition to citrullinated antigens, RA patients may generate antibodies against carbamylated, acetylated, and other post-translationally modified proteins (105). Anti-CarP antibodies are relevant because they may appear before clinical disease and have been associated with radiographic progression, including in some anti-CCP-negative patients (106). AAPAs add another layer of stratification and may occur independently of RF and ACPAs in some patients (105). However, AMPA interpretation is complicated by cross-reactivity, because some B-cell clones and antibodies can recognize multiple post-translational modifications, whereas others appear more modification-specific (107). Thus, AMPA profiling should not be viewed as a simple expansion of diagnostic markers, but as a way to assess the breadth, cross-reactivity, and maturation of autoreactive B-cell responses.

The relationship between ACPAs and bone provides a strong example of why autoantibody biology matters beyond diagnosis. ACPA-positive individuals may develop periarticular or systemic bone loss before clinically evident synovitis (95). Mechanistic studies suggest that antibodies against citrullinated vimentin can bind osteoclast-lineage cells, promote osteoclast differentiation, enhance bone-resorptive activity, and induce bone loss in experimental models (108). Nevertheless, this effect should not be generalized to all ACPAs. Different monoclonal ACPAs may vary in their ability to induce pain, tenosynovitis, osteoclastogenesis, or inflammatory cytokine production (109, 110). ACPA-associated bone damage is therefore better framed as clone- and context-dependent rather than as an automatic consequence of ACPA positivity.

Taken together, ACPA and AMPA heterogeneity provides a more precise framework for understanding why RA patients with the same clinical diagnosis may differ in disease onset, mucosal origin, systemic inflammation, bone erosion, and treatment response. Future stratification should move beyond simple anti-CCP positivity and incorporate multidimensional autoantibody profiling, including fine specificity, epitope spreading, citrullinome mapping, isotype distribution, Fab/Fc glycosylation, AMPA breadth, cross-reactivity, and osteoclast-related effector function. Such an approach may better align serological biomarkers with the mechanistic heterogeneity that contributes to RA progression and treatment non-response.

### 3.4 Cross-amplification of cytokine networks and core signaling pathways

Following innate and adaptive immune activation, RA inflammation is maintained by reciprocal amplification between cytokine networks and intracellular signaling pathways (111). Rather than depending on a single inflammatory mediator, the synovial microenvironment is shaped by interacting cytokines, receptor signaling, transcriptional programs, and impaired negative regulation. This network structure helps explain both disease chronicity and heterogeneous responses to targeted therapy (112).

TNF-α, IL-6, IL-1β, IL-17, IL-12, and IL-23 form major cytokine axes involved in RA synovitis (111). TNF-α and IL-1β contribute to local inflammatory activation and tissue-destructive programs (48, 79). IL-6 supports systemic inflammation, B-cell activation, Th17 polarization, and acute-phase responses (113), whereas IL-17, IL-12, and IL-23 help sustain pathogenic T-cell programs (91). These cytokines interact through shared downstream pathways. For example, TNF-α can induce IL-6 expression through NF-κB activation, while IL-6 may amplify inflammatory transcription through JAK/STAT3 signaling (79, 111). IL-17 can further enhance IL-6 production and reinforce inflammatory activation through overlapping NF-κB- and STAT3-related mechanisms (114).

The stability of this cytokine circuitry depends on cross-talk among NF-κB, JAK/STAT, and MAPK signaling. NF-κB is activated by TNF-α, TLR ligands, and ICs, and promotes the transcription of cytokines, chemokines, adhesion molecules, and MMPs (115). JAK/STAT signaling transduces multiple cytokine signals, particularly IL-6-related pathways, and influences pathogenic T-cell differentiation and FLS activation (116). MAPK cascades, including ERK, p38, and JNK branches, regulate cell proliferation, stress responses, and inflammatory gene expression (79). In the RA synovium, these pathways are not isolated modules but overlapping signaling circuits that can reinforce one another.

Chronic inflammation is further supported by insufficient anti-inflammatory counter-regulation. Under physiological conditions, IL-10, TGF-β, and SOCS proteins limit excessive cytokine signaling. In RA, these regulatory mechanisms may be weakened by epigenetic changes, oxidative stress, altered receptor signaling, and dysregulated SOCS expression (116, 117). For example, oxidative stress-related pathways may interfere with IL-10 receptor signaling, while abnormal SOCS3 activity may disrupt the balance between inflammatory and regulatory cytokine responses. This imbalance does not imply complete loss of immune regulation, but it may reduce the capacity of the synovial microenvironment to resolve inflammation.

**Figure 3:**
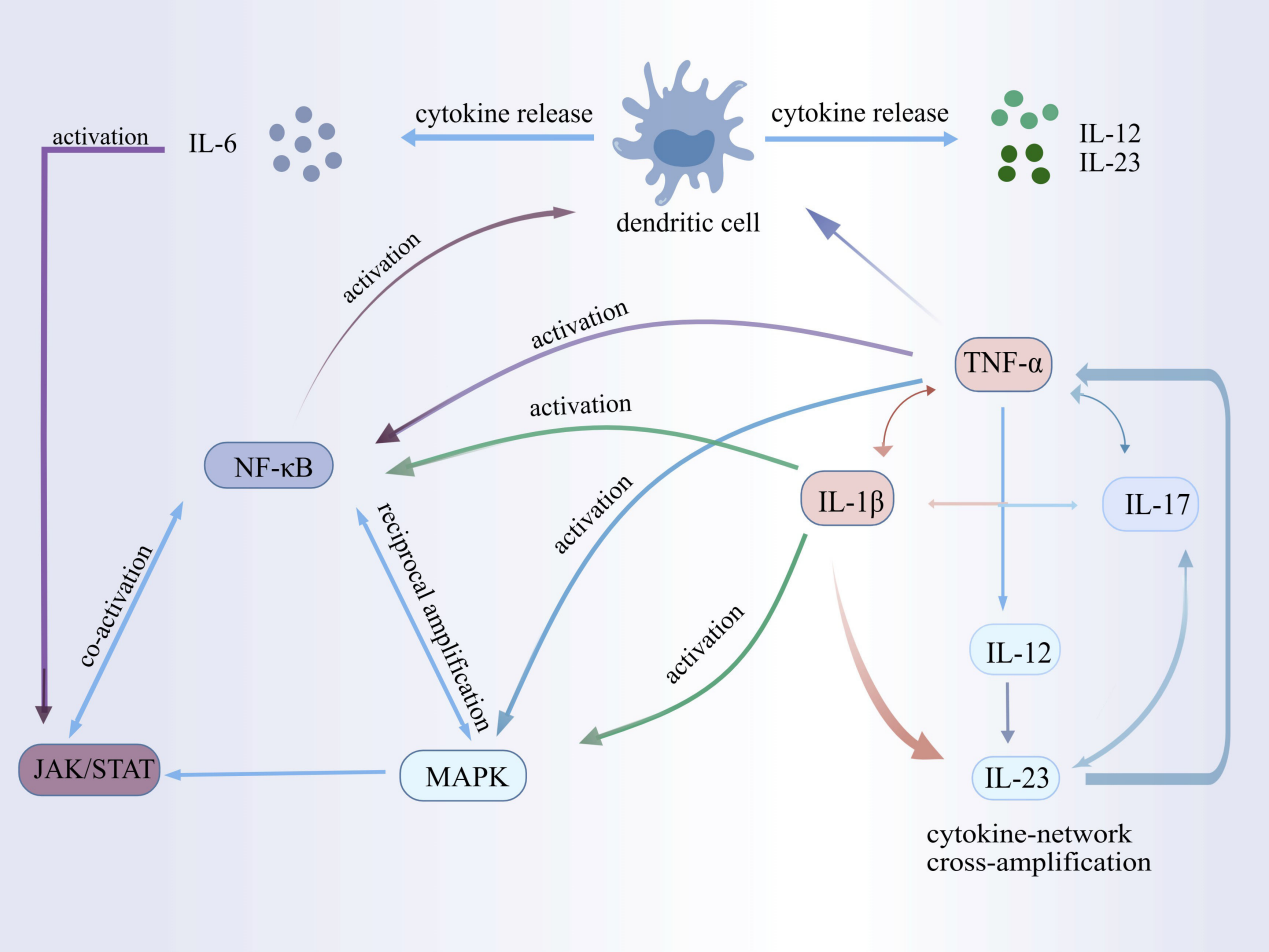
Cytokine-network cross-amplification and core signaling pathways in RA. TNF-α, IL-6, IL-1β, IL-17, IL-12, and IL-23 participate in overlapping cytokine and signaling circuits that activate NF-κB, JAK/STAT, and MAPK signaling. Reciprocal amplification among these cytokines and pathways may sustain synovial inflammation, promote immune-cell and stromal-cell activation, and contribute to treatment-response variability.

Importantly, cytokine and signaling networks vary across patients and disease stages. Some patients may show a TNF-α/NF-κB-dominant profile, whereas others may depend more heavily on IL-6/JAK/STAT3, IL-17-related signaling, interferon-associated programs, or mixed inflammatory circuits (112, 118). This pathway dependence may partly explain why TNF inhibitors, IL-6 receptor blockade, JAK inhibitors, or other targeted therapies produce different responses among clinically similar patients. Non-response may occur when the targeted pathway is not the dominant driver in the current disease state, or when compensatory signaling emerges under therapeutic pressure. Therefore, cytokine-network cross-amplification represents a key mechanism linking RA immune heterogeneity with chronic synovitis, structural progression, and treatment-response variability (**Figure 3**).

### 3.5 Synovial pathogenic niche remodeling and joint tissue destruction

With persistent innate immune activation, adaptive immune amplification, and cytokine-network cross-talk, RA pathology gradually shifts from systemic immune dysregulation toward local remodeling of the synovial microenvironment (119). This pathogenic niche contains infiltrating leukocytes, activated FLS, angiogenic endothelial cells, macrophages, and osteoclast-lineage cells (120). Through reciprocal immune–stromal interactions, the synovium can become a self-sustaining site of chronic inflammation, tissue invasion, and structural damage (121).

FLS are major effector cells in this process. Under physiological conditions, they help maintain synovial architecture and joint-fluid homeostasis (122). In RA, continuous exposure to TNF-α, IL-1β, IL-6, IL-17, hypoxia, and matrix-derived signals may induce an aggressive FLS phenotype characterized by proliferation, migration, invasion, apoptosis resistance, and increased production of chemokines and inflammatory mediators (122, 123). Activated FLS also produce MMP-1, MMP-3, and MMP-13, which contribute to degradation of cartilage matrix components (124). This phenotypic shift allows FLS to function not only as passive stromal cells but also as active participants in leukocyte recruitment, matrix remodeling, and tissue destruction.

**Figure 4:**
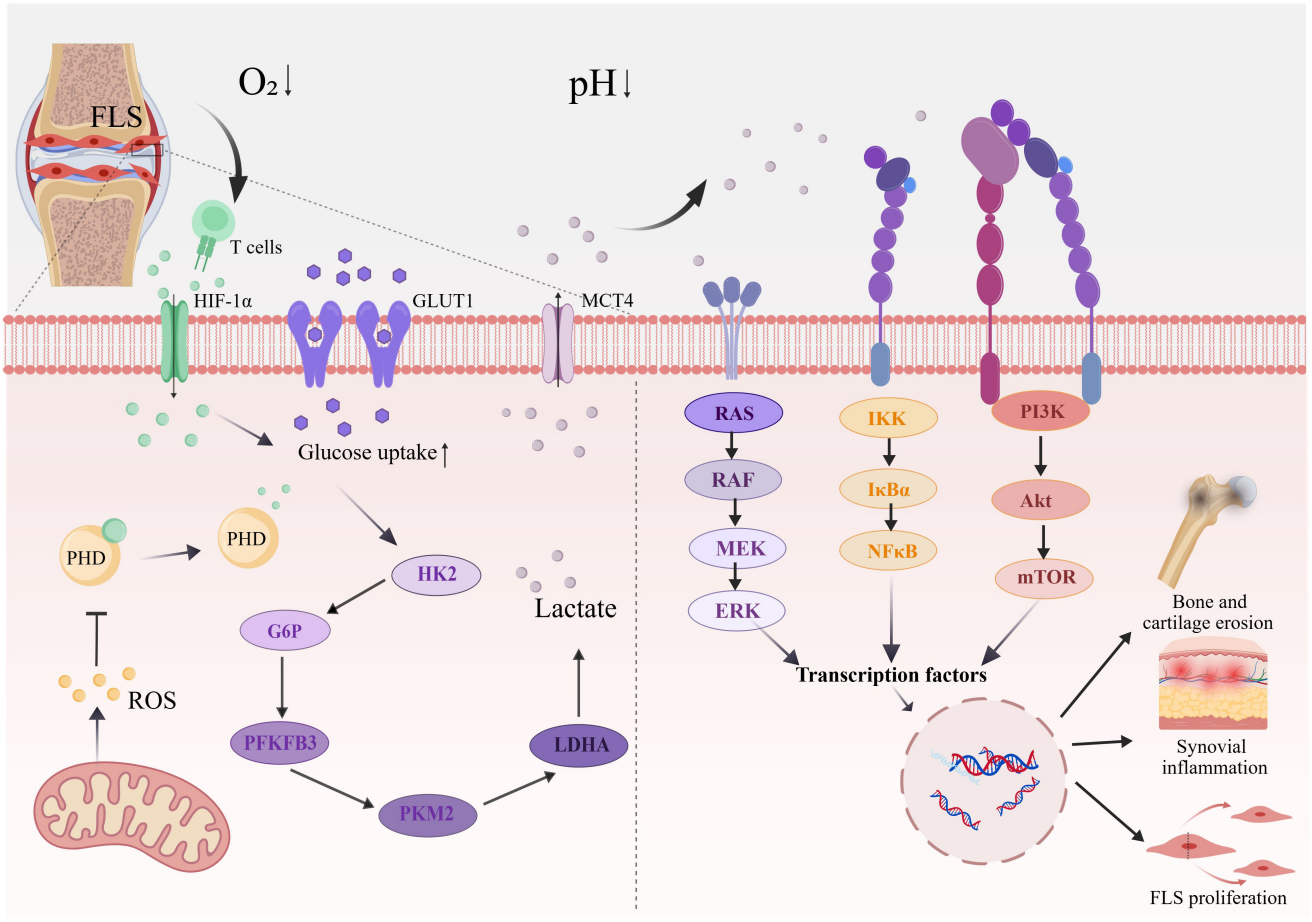
Metabolic and inflammatory remodeling of the RA synovial niche. Abnormal angiogenesis, hypoxia, acidosis, oxidative stress, and altered glycolytic metabolism may reshape the RA synovial niche. Increased glucose uptake and lactate production can interact with hypoxia-associated signaling, ROS accumulation, and inflammatory pathways such as NF-κB and MAPK-related signaling, thereby promoting FLS activation, inflammatory amplification, FLS proliferation, cartilage degradation, and bone erosion.

Pathological angiogenesis further supports the synovial niche. Inflammatory mediators upregulate angiogenic factors and adhesion molecules, promoting endothelial activation, vascular permeability, and leukocyte entry into synovial tissue (125, 126). However, the newly formed microvasculature is often functionally abnormal and may coexist with local hypoxia, acidosis, and metabolic stress. These conditions can reinforce FLS activation, macrophage inflammatory programs, and tissue-remodeling responses (127) (**Figure 4**).

**Figure 5:**
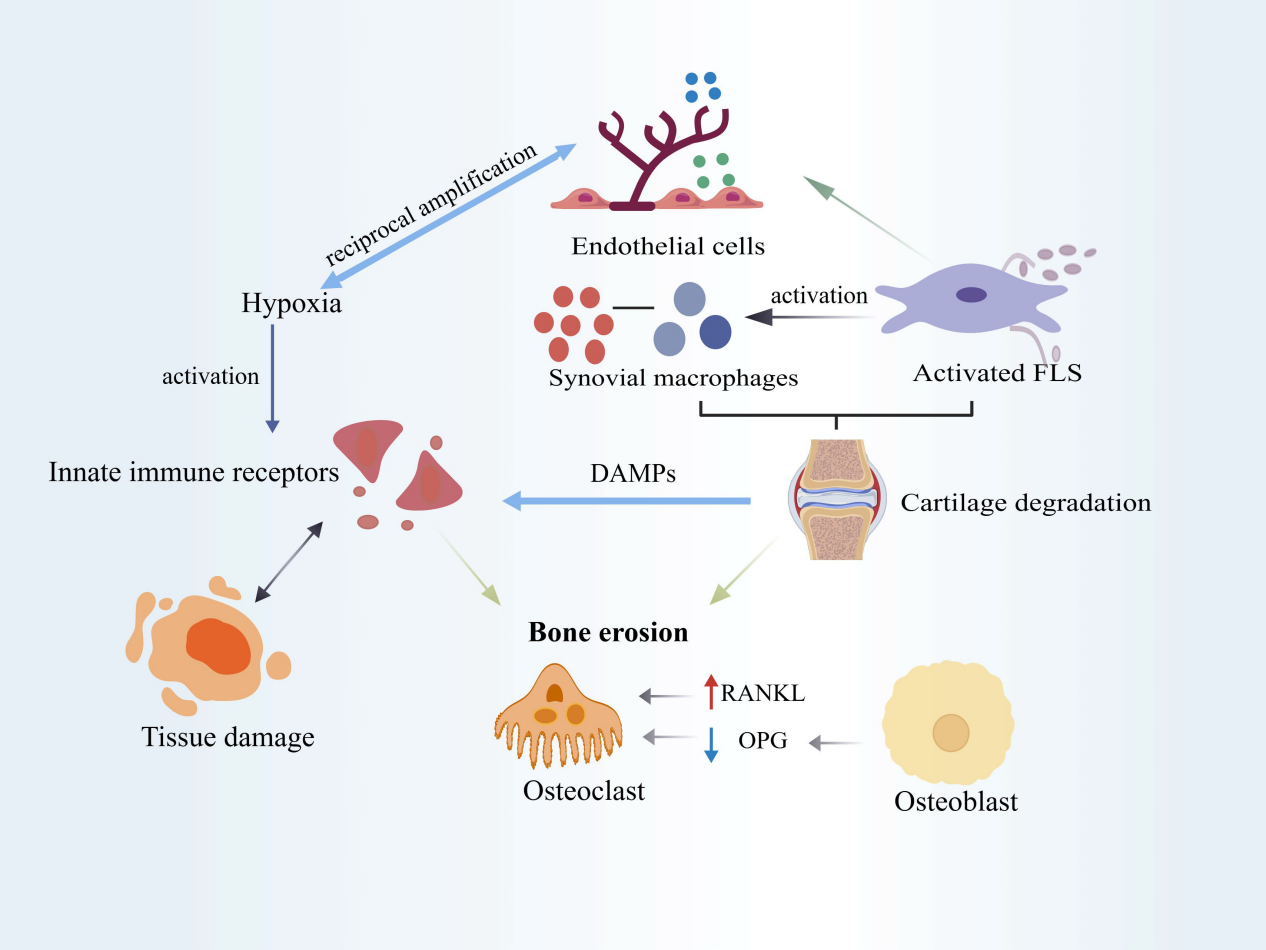
Synovial pathogenic niche remodeling and joint tissue destruction in RA. Hypoxia, abnormal angiogenesis, activated FLS, synovial macrophages, DAMPs, and innate immune activation contribute to a self-sustaining inflammatory synovial niche. Immune–stromal crosstalk promotes cartilage degradation and shifts the RANKL/OPG balance toward osteoclastogenesis, thereby driving bone erosion and structural damage.

Cartilage erosion and bone destruction represent major structural consequences of sustained synovial inflammation. Cartilage damage is mediated in part by MMPs released by FLS and macrophages, which degrade type II collagen and proteoglycans (128). Bone erosion is closely linked to osteoclastogenesis. Inflammatory cytokines and activated immune–stromal interactions promote RANKL expression while reducing OPG-mediated restraint, thereby shifting the RANKL/OPG balance toward osteoclast differentiation and bone resorption (129). This process links immune activation with structural progression, although the magnitude of bone damage varies across patients and disease stages (**Figure 5**).

The synovial microenvironment can also amplify inflammation through feedback mechanisms. FLS-derived chemokines promote local retention of immune cells; matrix fragments generated during cartilage degradation may act as endogenous danger signals; and hypoxia-associated metabolic remodeling may reinforce inflammatory and invasive stromal programs (127). These circuits may help explain why local joint damage can progress even when systemic inflammatory markers decline in some patients.

**Figure 6:**
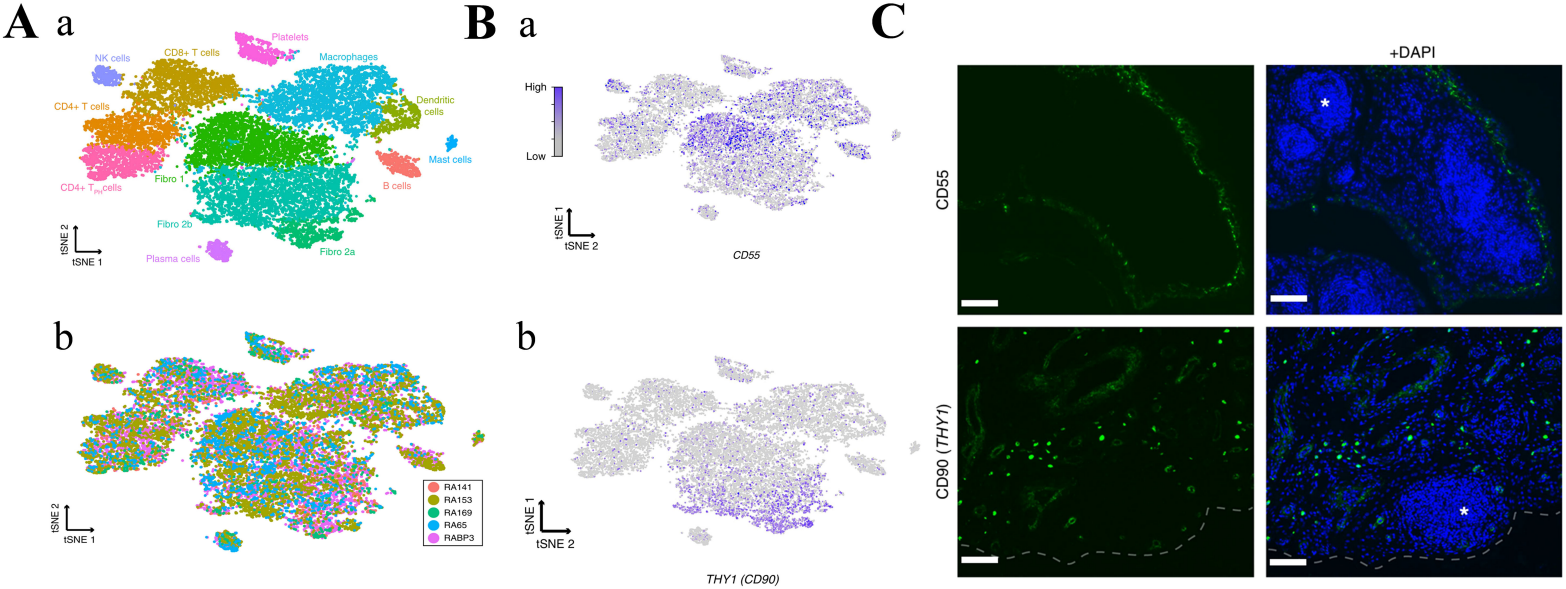
Single-cell evidence for synovial immune and stromal cell heterogeneity in RA. **(A)** Single-cell transcriptomic clustering of RA synovial tissue identifies multiple immune and stromal cell populations, including T cells, B cells, plasma cells, macrophages, dendritic cells, mast cells, fibroblast subsets, endothelial cells, and platelets. **(B)** Expression patterns of CD55 and THY1/CD90 highlight distinct fibroblast-associated transcriptional states within the RA synovial single-cell landscape. **(C)** Immunofluorescence staining shows the spatial distribution of CD55-positive and CD90/THY1-positive stromal populations in RA synovial tissue, supporting regional organization of fibroblast subsets. Adapted and summarized from ref (132, 133).

From a translational perspective, synovial remodeling provides a biological basis for tissue-level stratification. Synovial pathotypes, including lympho-myeloid, diffuse-myeloid, and pauci-immune/fibroid patterns, reflect differences in cellular architecture and dominant inflammatory or stromal programs (81). However, their predictive value for treatment selection should be interpreted cautiously and is discussed in detail in Section 5.2. At present, direct therapeutic targeting of FLS or the synovial microenvironment remains investigational because of limited disease-specific stromal markers, delivery barriers, and potential off-target effects (**Table 2**). Therefore, synovial niche remodeling should be viewed as a key mechanism of joint damage and a promising but still developing basis for precision intervention (**Figure 6**).

**Table 2:** Pathogenic immune networks, synovial pathotypes, and tissue-destructive programs in RA.

Pathogenic module	Key cells or mediators	Dominant mechanisms	Related synovial pathotype	Tissue consequence	Therapeutic relevance	References
Innate immune activation	Macrophages, DCs, neutrophils, mast cells, DAMPs, ICs	Danger-signal sensing, antigen presentation, cytokine release, leukocyte recruitment, and early inflammatory amplification	More evident in diffuse-myeloid synovial pathotype, but may also contribute to mixed inflammatory synovitis	Initiation and amplification of synovitis, local inflammatory-cell infiltration, and transition toward adaptive immune activation	Supports therapeutic targeting of upstream inflammatory cytokines and innate immune amplification pathways	(76, 77)
Synovial macrophage state remodeling	Inflammatory macrophage states, MERTK-positive resident macrophages, TNF-α, IL-6, IL-1β, CXCL chemokines, SPP1, SLAMF7	Expansion of inflammatory macrophage programs, myeloid–stromal crosstalk, and imbalance between inflammatory and inflammation-resolving macrophage states	Closely associated with diffuse-myeloid synovial pathotype; macrophage-rich states may also coexist with lympho-myeloid synovial pathotype	Persistent cytokine production, endothelial activation, leukocyte recruitment, and inflammatory niche stabilization	May help explain variable responses to TNF-α blockade, IL-6 receptor blockade, and JAK inhibition; macrophage-state profiling remains investigational	(122, 124)
DC-mediated T-cell activation	DCs, CD4+ T cells, Th1 cells, Th17 cells, Tfh cells, co-stimulatory signals, IL-12, IL-23, IL-21	Enhanced antigen presentation, co-stimulatory signaling, and differentiation of pathogenic T-cell programs	Frequently linked to lympho-myeloid synovial pathotype, especially when lymphoid aggregates or TLS-like organization are present	Sustained adaptive immune activation, T-cell help for B cells, cytokine amplification, and autoantibody-supporting immune organization	Provides rationale for T-cell co-stimulation blockade and for interpreting T-cell-dominant or Tfh–B-cell-associated disease programs	(64, 130)
Th17-skewed inflammatory axis	Th17 cells, IL-17, IL-6, IL-23, neutrophils, FLS, osteoclast-lineage cells	Th17 polarization, IL-17-associated cytokine amplification, neutrophil recruitment, FLS activation, and osteoclastogenesis	May occur across inflammatory synovial pathotypes; often overlaps with lympho-myeloid or diffuse-myeloid synovial pathotype	Increased synovial inflammation, MMP expression, cartilage degradation, and bone erosion	May inform pathway-level interpretation of IL-6/JAK/STAT-related activity and inflammatory amplification, although direct IL-17 targeting has limited established use in RA	(95, 122)
B-cell and humoral immune amplification	B cells, plasma cells, RF, ACPAs, ICs, complement, TLS	Autoantibody production, IC formation, complement activation, local antigen presentation, and in situ humoral immune responses	Most closely related to lympho-myeloid synovial pathotype	Local immune-complex-driven inflammation, macrophage and neutrophil activation, and persistence of organized synovial immunity	Supports consideration of B-cell depletion or T-cell co-stimulation blockade in selected humoral immune-dominant contexts, while requiring evidence-weighted interpretation	(7, 13)
ACPA/AMPA diversification	ACPAs, AMPAs, anti-CarP antibodies, AAPAs, ACPA fine specificity, Fab/Fc glycosylation	Epitope spreading, antibody cross-reactivity, altered effector function, IC formation, and context-dependent activation of macrophages or osteoclast-lineage cells	Often associated with seropositive RA and lympho-myeloid synovial pathotype, but autoantibody effects may occur outside a single tissue pattern	Increased risk of systemic autoimmunity, inflammatory amplification, pain, osteoclastogenesis, and structural damage in selected antibody profiles	Supports multidimensional serological stratification beyond anti-CCP positivity; may help refine erosion-risk assessment and treatment-response prediction	(7, 131)
Cytokine-network cross-amplification	TNF-α, IL-6, IL-1β, IL-17, IL-12, IL-23, NF-κB, JAK/STAT, MAPK, SOCS	Reciprocal cytokine induction, overlapping intracellular signaling, impaired negative regulation, and compensatory pathway activation	Can be present across pathotypes; TNF-α/NF-κB- or IL-6/JAK/STAT-dominant activity may be more apparent in myeloid-rich synovitis	Chronic synovitis, systemic inflammatory burden, persistent leukocyte recruitment, and treatment-response heterogeneity	Provides mechanistic basis for TNF inhibitors, IL-6 receptor blockade, JAK inhibitors, and treatment switching when pathway dependence changes	(113, 116)
FLS-driven stromal activation	FLS, chemokines, MMP-1, MMP-3, MMP-13, hypoxia-related signals, matrix-derived cues	FLS proliferation, migration, invasion, apoptosis resistance, chemokine production, and matrix-degrading program activation	Prominent in pauci-immune/fibroid synovial pathotype; may also contribute to refractory tissue-embedded inflammation in mixed pathotypes	Pannus formation, cartilage invasion, extracellular matrix degradation, and persistence of local tissue pathology	Suggests that stromal-dominant disease may respond poorly to conventional cytokine-directed strategies; FLS- or niche-targeted approaches remain investigational	(122, 123)
Angiogenic and metabolic synovial niche remodeling	Endothelial cells, angiogenic mediators, hypoxia, acidosis, metabolic stress, macrophages, FLS	Abnormal angiogenesis, increased vascular permeability, leukocyte entry, local hypoxia, and metabolic reinforcement of inflammatory stromal programs	May accompany lympho-myeloid and diffuse-myeloid synovial pathotypes; stromal remodeling may become more dominant in pauci-immune/fibroid synovial pathotype	Formation of a self-sustaining inflammatory niche, enhanced immune-cell retention, and persistence of synovial inflammation	Supports local microenvironment-oriented therapeutic concepts, including targeted delivery and stromal or metabolic modulation in research settings	(125–127)
Osteoclastogenesis and structural damage	RANKL/OPG axis, osteoclast-lineage cells, FLS, macrophages, ACPAs, MMPs	Increased RANKL expression, reduced OPG-mediated restraint, osteoclast differentiation, cartilage matrix degradation, and antibody-associated bone effects	Can occur across active inflammatory synovial pathotypes; risk may be enhanced in seropositive and tissue-destructive disease states	Bone erosion, cartilage destruction, joint-space narrowing, and irreversible structural progression	Supports early identification of high-risk structural progression and reinforces the need to align anti-inflammatory treatment with tissue-damage monitoring	(128, 129)
Integrated tissue-destructive microenvironment	Macrophages, DCs, T cells, B cells, FLS, endothelial cells, osteoclasts, cytokines, autoantibodies	Reciprocal immune–stromal crosstalk, cytokine-network reinforcement, autoantibody-driven amplification, angiogenesis, and matrix remodeling	Reflects the combined output of lympho-myeloid, diffuse-myeloid, and pauci-immune/fibroid synovial pathotypes	Persistent synovitis, pannus formation, cartilage destruction, bone erosion, and heterogeneous therapeutic response	Supports synovial pathotype-based stratification and mechanism-informed treatment selection, but clinical implementation requires further validation	(120, 121)

## 4 Gut-joint axis-related immune-metabolic amplification and systemic heterogeneity

RA progression is not confined to the synovial immune network. Increasing evidence suggests that mucosal immune dysregulation, intestinal barrier dysfunction, gut microbiome alterations, and microbial metabolite changes may contribute to systemic immune-metabolic heterogeneity in RA (134). As a major immune interface, the gut can influence host immunity through microbial products and metabolites, including short-chain fatty acids (SCFAs), tryptophan metabolites, and secondary bile acids. These mediators may regulate T-cell differentiation, myeloid-cell activity, autoantibody-related immune responses, and inflammatory tone in a direct or indirect manner (135). Therefore, the gut-joint axis should be viewed as a potential cross-organ regulatory network linking environmental exposure, mucosal immune imbalance, systemic inflammation, and joint pathology, rather than as a proven universal causal pathway (136).

The gut-joint axis may operate bidirectionally during RA development and progression. Gut microbiome alterations and metabolite imbalance may influence mucosal tolerance, Th17-skewed inflammation, systemic cytokine production, and autoantibody-associated immune activation. Conversely, chronic joint inflammation, oxidative stress, inflammatory cytokines, medication exposure, and altered host metabolism may reshape the intestinal microenvironment and further affect microbial composition and barrier integrity (137). This reciprocal interaction may help explain why RA patients differ in microbiome composition, metabolic phenotype, inflammatory persistence, and treatment response across disease stages and therapeutic backgrounds (138, 139). However, most human evidence remains associative, and causality is difficult to establish because microbiome profiles are shaped by diet, geography, medication, disease activity, and sampling methods. Thus, the gut-joint axis provides an important but still evolving framework for understanding RA heterogeneity and may offer adjunctive biomarkers or therapeutic targets after further longitudinal and interventional validation (140).

### 4.1 Gut microbial imbalance, P. copri, and mucosal immune activation

Gut microbial imbalance has been implicated as a potential contributor to RA-related mucosal immune dysregulation, although its causal position relative to genetic susceptibility, environmental exposure, and preclinical autoimmunity remains incompletely defined (141). The gut functions as a major mucosal immune interface in which epithelial barrier integrity, commensal microbes, microbial products, and local immune cells cooperate to maintain immune regulation (142). In RA, this balance may be disturbed by expansion of selected pathobionts, reduced commensal diversity, altered microbial functional capacity, and impaired epithelial barrier integrity (106). These changes may increase mucosal immune activation and systemic inflammatory tone, but their clinical significance is likely to vary across patient subsets.

Among candidate microbial taxa, Prevotella, particularly P. copri, has attracted substantial attention. Several studies have reported enrichment of P. copri in subsets of patients with new-onset or untreated RA and in some individuals at risk of RA (143, 144). Mechanistic studies suggest that RA-associated P. copri strains or P. copri-dominant microbiota may enhance mucosal immune activation and exacerbate arthritis in susceptible animal models (145). Immune-reactivity studies have also identified antibody and T-cell responses to P. copri-derived antigens in subsets of RA patients, suggesting a possible link between microbial antigen exposure and systemic autoimmunity (146, 147).

Nevertheless, the causal role of P. copri in human RA remains unresolved. Most human microbiome studies are cross-sectional or observational, making it difficult to determine whether P. copri expansion precedes RA, reflects early inflammation, tracks with treatment status, or is shaped by diet, geography, and medication exposure. P. copri is also not uniformly pathogenic; it can be abundant in healthy individuals, and strain-level analyses indicate substantial genomic and functional heterogeneity between RA-derived and healthy-control-derived isolates (148). Therefore, P. copri should be framed as a context-dependent microbial candidate that may contribute to RA in genetically and immunologically susceptible hosts, rather than as a proven universal causal agent.

From a translational perspective, gut microbiome profiling may provide adjunctive information for risk assessment or phenotype stratification, but it currently lacks sufficient specificity and reproducibility for routine diagnostic use (140). Its clinical implementation remains limited by inter-individual variability, dietary and geographic confounding, medication effects, inconsistent sampling methods, and insufficient longitudinal validation (149, 150). Thus, microbiome composition should be viewed as one exploratory layer of RA heterogeneity rather than as an independent clinical decision tool.

### 4.2 Microbial metabolites and immune-metabolic remodeling

Microbial metabolites provide a functional link between gut microbiome alterations, mucosal immune activity, and systemic inflammation in RA. Unlike taxonomic profiling, which describes microbial composition, metabolite analysis may better reflect the biological output of the microbiome. Alterations in SCFAs, tryptophan metabolites, secondary bile acids, lactate, and oxidative lipid products may influence epithelial barrier integrity, myeloid-cell activation, systemic inflammatory tone, and synovial immune-metabolic states (151). These changes should not be interpreted as independent disease drivers, but rather as components of a broader immune-metabolic network shaped by microbiome composition, diet, systemic inflammation, medication exposure, and disease stage (137, 152).

SCFAs, particularly butyrate and propionate, are relevant because they can support epithelial barrier function and contribute to mucosal immune regulation (113, 153). Reduced abundance of SCFA-producing commensals may therefore weaken barrier stability and increase mucosal exposure to microbial products. Tryptophan-derived metabolites may influence immune regulation through AhR-related pathways, whereas altered secondary bile acid metabolism may affect myeloid-cell activation, inflammatory signaling, and osteoclast-related pathways (154). In parallel, lactate accumulation, oxidative stress, and lipid remodeling may reinforce inflammatory programs in circulating immune cells and synovial tissue (155, 156).

These metabolic changes may help explain why patients with similar clinical diagnoses can differ in systemic inflammatory tone, synovial activation, and treatment response. However, the direction and magnitude of metabolite-associated effects are likely to vary across patient subsets and tissue compartments. In addition, metabolomic signatures are highly sensitive to diet, medication exposure, sample type, analytical platform, and disease activity, which limits direct clinical interpretation (135).

**Figure 7:**
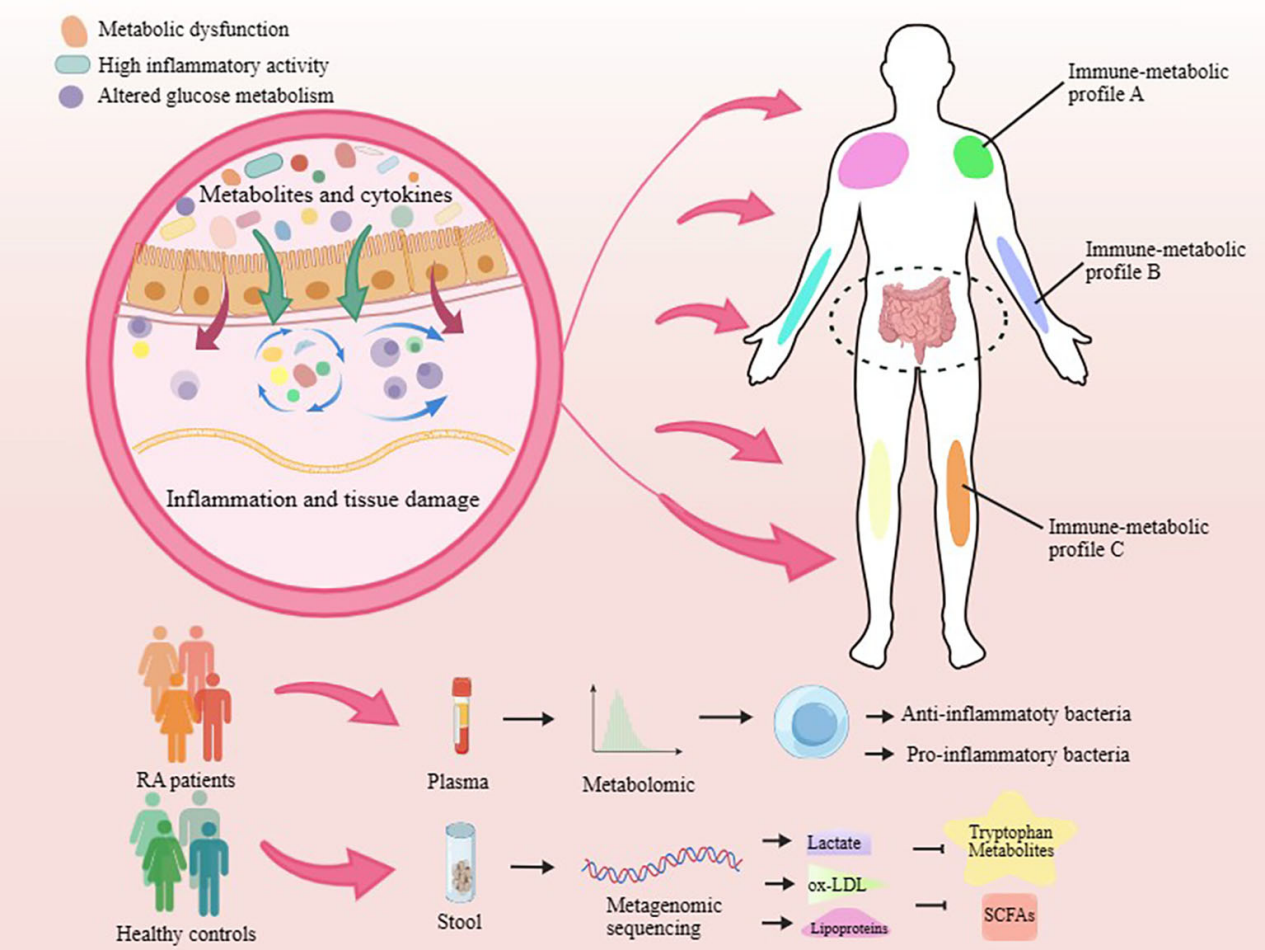
Gut-joint axis-related microbiome-metabolome interactions and systemic immune-metabolic heterogeneity in RA. Gut microbial imbalance may contribute to systemic immune-metabolic heterogeneity in RA through microbial metabolite changes, intestinal barrier dysfunction, and mucosal immune dysregulation. Plasma metabolomics and stool metagenomic sequencing may provide complementary information on metabolic phenotype, systemic inflammatory tone, altered glucose metabolism, tryptophan metabolites, SCFAs, and lipid remodeling. These gut-derived and systemic metabolic signals may help define distinct immune-metabolic profiles, but their clinical use remains exploratory and requires standardized sampling, longitudinal validation, and integration with serology, imaging, and synovial molecular pathology.

From a translational perspective, metabolomic profiling may provide adjunctive biomarkers for disease activity, structural progression, and treatment-response prediction (129, 157). When integrated with microbiome data, synovial molecular pathology, imaging, and longitudinal clinical outcomes, metabolic signatures may help define immune-metabolic RA profiles (**Figure 7**). Nevertheless, metabolic biomarkers remain exploratory and require standardized sampling, external validation, and prospective testing before they can be used for routine stratification or treatment selection.

### 4.3 Gut microbial and metabolic regulation of the Th17/Treg axis

The Th17/Treg axis provides an important immunological bridge between gut microbial imbalance, metabolic remodeling, and systemic inflammation in RA (119). Under physiological conditions, Th17 cells contribute to mucosal defense, whereas Tregs restrain excessive inflammation and help maintain immune regulation. In RA, altered microbial composition, reduced immunoregulatory metabolites, and impaired barrier integrity may shift this balance toward Th17-skewed inflammation and weakened regulatory control (158).

SCFAs are among the microbial metabolites most closely linked to this axis. Butyrate and related metabolites may promote FOXP3 expression, enhance Treg stability, and limit excessive inflammatory cytokine production (153). Reduced SCFA-related signaling may therefore weaken mucosal regulatory programs. In parallel, altered tryptophan metabolism may reduce AhR-mediated regulatory signaling, thereby favoring Th17 activation and IL-17-associated inflammation (154). These mechanisms provide a plausible explanation for how gut microbial and metabolic changes may influence immune polarization, although their relative contribution in human RA remains variable across cohorts (159).

Once established, Th17-skewed immune responses may contribute to systemic and synovial inflammation. Th17-associated mediators, particularly IL-17, can promote chemokine production, neutrophil recruitment, FLS activation, MMPs expression, and osteoclastogenesis (160, 161). At the same time, insufficient Treg-mediated regulation may reduce control over autoreactive T cells, B cells, and myeloid cells, thereby facilitating broader immune amplification (162). However, this process should be interpreted as one component of RA immune heterogeneity rather than as a universal causal pathway (163).

Clinically, Th17/Treg-associated markers may provide adjunctive information for risk assessment and phenotype stratification, especially when interpreted together with microbiome, metabolomic, serological, imaging, and synovial molecular data (164). Interventions such as probiotics, butyrate supplementation, or metabolite-directed strategies remain investigational and require patient stratification, standardized endpoints, and longitudinal validation (165, 166). Thus, the Th17/Treg axis is best viewed as a mechanistic bridge linking gut-joint axis biology with RA immune heterogeneity, rather than as an independently validated therapeutic target.

### 4.4 Clinical positioning of gut-joint axis-based stratification and adjunctive intervention

The clinical translation of gut-joint axis research requires a realistic assessment of diagnostic limitations and adjunctive efficacy. Routine fecal microbiome profiling currently provides limited incremental value over established serological markers, imaging, and clinical assessment for baseline RA diagnosis (136, 167). Substantial inter-individual variability, dietary and geographic confounding, medication effects, and overlapping dysbiotic patterns across immune-mediated diseases limit its use as a primary diagnostic tool (168). Its more plausible clinical role is as an adjunctive stratification approach, particularly in pharmacomicrobiomics, where baseline microbial composition may influence MTX metabolism, bioavailability, enterohepatic circulation, and gastrointestinal toxicity risk (169, 170).

Microbiome-targeted interventions also require cautious interpretation. Probiotics, prebiotics, dietary modification, and metabolite supplementation have shown potential anti-inflammatory or barrier-supporting effects in experimental and small clinical studies, but current evidence does not support their use as stand-alone therapies capable of inducing sustained remission or preventing structural progression. Their effects are likely modest, context-dependent, and influenced by baseline microbiome composition, intestinal inflammation, medication exposure, and host immune status (138, 140). More invasive approaches, such as fecal microbiota transplantation, remain exploratory because of uncertainties regarding donor selection, engraftment durability, safety, and long-term immune effects (171).

A more clinically feasible strategy is to identify patient subgroups in whom gut-directed adjunctive approaches may be biologically justified. Candidate groups include individuals in a preclinical or early seropositive phase with evidence of barrier dysfunction or mucosal autoimmunity (172); patients with MTX intolerance or recurrent gastrointestinal toxicity, in whom microbial metabolism and bile acid dysregulation may affect drug exposure (173) and adherence; and refractory RA patients with metabolic syndrome, intestinal barrier impairment, or inflammatory bowel disease-like dysbiosis, where chronic endotoxin exposure may reinforce systemic and synovial inflammation (174). These subgroup hypotheses remain to be validated prospectively (167), but they provide a more precise framework than non-specific microbiome modulation.

**Figure 8:**
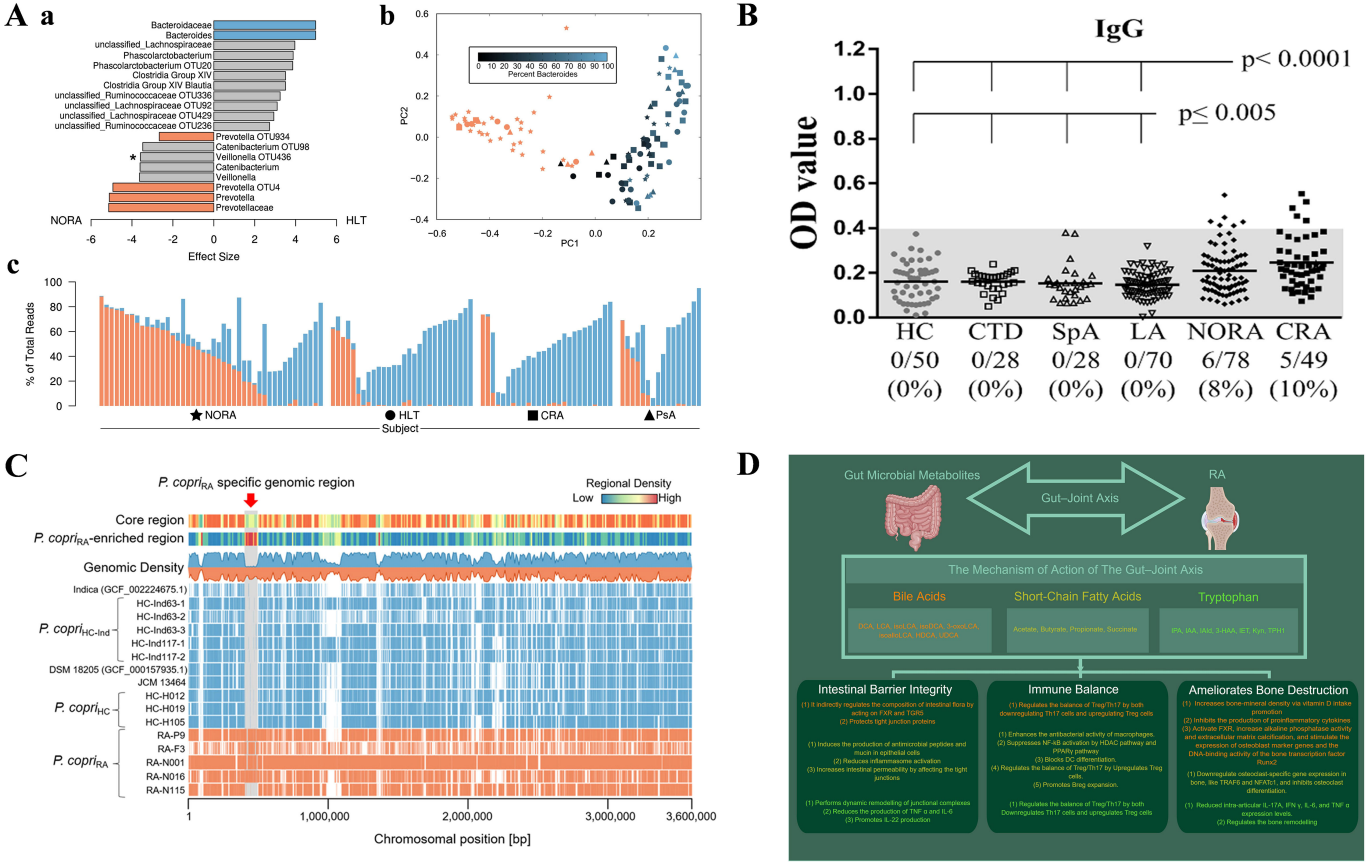
Evidence map and translational positioning of the gut-joint axis in RA. **(A)** Gut microbial profiling shows *Prevotella*-dominant microbial signatures in new-onset untreated RA. **(B)** IgG responses to whole *P. copri* are detected in subsets of new-onset and chronic RA, supporting its immune relevance in selected patients. **(C)** Genomic comparison of RA- and healthy control-derived *P. copri* strains highlights strain-level heterogeneity and RA-associated genomic features. **(D)** Gut microbial metabolites, including bile acids, SCFAs, and tryptophan metabolites, may link microbiome alterations with barrier integrity, immune regulation, systemic inflammation, and bone remodeling. Adapted and summarized from refs (103, 146, 148).

Overall, gut-joint axis-based strategies should currently be positioned as adjunctive tools for risk assessment, treatment-tolerance prediction, and mechanistic subtyping rather than as established disease-modifying interventions (**Figure 8**). Future clinical implementation will require standardized sampling, longitudinal microbiome-metabolome profiling, defined patient subgroups, validated endpoints, and integration with conventional clinical, serological, imaging, and synovial molecular data (**Table 3**).

**Table 3:** Evidence, limitations, and clinical positioning of gut-joint axis-related stratification in RA.

Gut-joint axis component	Main evidence	Potential mechanism	Candidate patient subgroup	Translational value	Current limitation	References
Gut microbial imbalance	Altered gut microbial diversity, expansion of selected pathobionts, reduced commensal abundance, and altered microbial functional capacity have been reported in RA and at-risk individuals	May increase mucosal immune activation, impair epithelial barrier regulation, and contribute to systemic inflammatory tone	Patients with early RA, preclinical autoimmunity, seropositive disease, or discordant inflammatory activity	May provide an exploratory layer for risk assessment and phenotype stratification when integrated with serology, imaging, and synovial molecular data	Mostly associative evidence; findings are affected by diet, geography, medication exposure, sampling methods, and inter-individual variability	(109, 110, 115)
P. copri-related microbial signatures	Enrichment of P. copri has been reported in subsets of new-onset, untreated, or at-risk RA patients; immune responses to P. copri-derived antigens have been detected in selected RA subsets	May promote mucosal immune activation, microbial antigen exposure, and systemic autoimmunity in genetically or immunologically susceptible hosts	New-onset or untreated RA; individuals at risk of RA; selected seropositive patients with mucosal immune features	May help define a microbiome-associated RA profile, but should be interpreted as a context-dependent microbial candidate rather than a universal causal agent	Human evidence remains largely cross-sectional; P. copri is also present in healthy individuals, and strain-level heterogeneity limits causal interpretation	(109, 145, 146)
Intestinal barrier dysfunction	Barrier impairment and increased mucosal exposure to microbial products have been linked to RA-related immune dysregulation	May facilitate microbial product translocation, innate immune activation, Th17-skewed inflammation, and systemic inflammatory amplification	Patients with early seropositive RA, mucosal autoimmunity, gastrointestinal symptoms, metabolic comorbidity, or inflammatory bowel disease-like features	May help identify patients in whom gut-directed adjunctive strategies are biologically plausible	Barrier assays are not standardized for routine RA stratification, and causal direction between inflammation and barrier dysfunction remains unclear	(115, 147, 149)
SCFA-related metabolic regulation	Reduced abundance of SCFA-producing commensals and altered SCFA-related signaling have been associated with immune dysregulation in RA	SCFAs, particularly butyrate and propionate, may support epithelial barrier integrity, promote Treg stability, and restrain excessive inflammatory cytokine production	Patients with reduced SCFA-producing taxa, Th17/Treg imbalance, early inflammatory disease, or difficult-to-control systemic inflammation	May support metabolite-informed stratification and provide a rationale for dietary, prebiotic, probiotic, or butyrate-directed adjunctive studies	Effects are context-dependent; SCFA levels are influenced by diet, sampling, gut ecology, medication exposure, and host metabolism	(110, 137, 153)
Tryptophan metabolite–AhR signaling	Altered tryptophan metabolism and AhR-related immune regulation have been implicated in gut-mediated immune remodeling	May reduce AhR-mediated regulatory signaling and favor Th17 activation, IL-17-associated inflammation, and weakened mucosal immune regulation	Patients with Th17-skewed inflammation, reduced regulatory immune tone, or microbiome-metabolome abnormalities	May provide a mechanistic link between microbial metabolism and immune polarization	Direct clinical biomarkers and therapeutic thresholds are not established; evidence remains heterogeneous across cohorts	(110, 124, 154)
Secondary bile acid metabolism	Altered secondary bile acid metabolism has been linked to immune-metabolic remodeling and inflammatory regulation	May affect myeloid-cell activation, inflammatory signaling, osteoclast-related pathways, and systemic immune-metabolic tone	Patients with metabolic syndrome, altered gut-liver metabolism, intestinal dysbiosis, or difficult-to-treat RA	May contribute to immune-metabolic profiling and treatment-tolerance assessment	Sensitive to diet, liver function, medication exposure, microbiome composition, and analytical platform differences	(123, 124, 154)
Lactate, oxidative lipid products, and immune-metabolic remodeling	Lactate accumulation, oxidative stress, and lipid remodeling have been associated with inflammatory immune-cell and synovial programs	May reinforce inflammatory programs in circulating immune cells and synovial tissue and connect systemic metabolism with synovial inflammation	Patients with high inflammatory burden, metabolic comorbidity, persistent synovitis, or refractory disease	May help define immune-metabolic RA profiles when integrated with synovial pathology and longitudinal outcomes	Metabolomic signatures are highly platform-, diet-, medication-, and disease-activity-dependent	(128, 156, 175)
Th17/Treg axis	Gut microbial and metabolic alterations have been linked to Th17/Treg imbalance in RA	Reduced immunoregulatory metabolites and impaired barrier integrity may shift mucosal and systemic immunity toward Th17-skewed inflammation and weakened Treg-mediated control	Patients with early autoimmunity, seropositive RA, mucosal immune activation, or Th17-dominant inflammatory features	May serve as a mechanistic bridge linking gut-joint axis biology with RA immune heterogeneity	Should be viewed as one component of RA heterogeneity rather than a universal causal pathway or independently validated therapeutic target	(118, 163, 176)
Microbiome profiling	Fecal microbiome profiling can identify compositional and functional microbial differences in RA cohorts	May capture microbiome-associated immune-metabolic features that complement serology, imaging, and clinical phenotype	Seronegative or atypical RA, preclinical at-risk individuals, refractory RA, or patients with gastrointestinal/metabolic features	May provide adjunctive information for risk assessment, phenotype stratification, and research-based patient subgrouping	Insufficient specificity and reproducibility for routine diagnosis; strong confounding by diet, geography, medication, and sampling workflow	(106, 140, 149)
Pharmacometabolomics and pharmacomicrobiomics	Baseline microbial composition and microbial metabolism may influence MTX metabolism, bioavailability, enterohepatic circulation, and gastrointestinal toxicity	Gut microbial enzymes and metabolite profiles may alter drug exposure, tolerance, and adherence	Patients receiving MTX, patients with MTX intolerance, recurrent gastrointestinal toxicity, or variable drug exposure	May support treatment-tolerance prediction and individualized management of csDMARD therapy	Clinical implementation requires standardized microbiome-metabolome workflows and prospective validation	(169, 170)
Probiotics, prebiotics, and dietary modulation	Experimental and small clinical studies suggest potential anti-inflammatory or barrier-supporting effects	May increase beneficial commensals, restore metabolite balance, improve barrier function, and reduce inflammatory tone	Patients with early disease, mild gut-related features, reduced SCFA-producing taxa, or metabolic comorbidity	Potential adjunctive strategy to support immune-metabolic regulation	Effects are modest, context-dependent, and not established as disease-modifying therapy for RA	(138, 165, 166)
Butyrate or metabolite-directed supplementation	Butyrate and related metabolites may promote regulatory immune programs and barrier integrity in experimental or mechanistic contexts	May enhance Treg stability, restrain inflammatory cytokine production, and partially counteract Th17/Treg imbalance	Patients with reduced SCFA-related signatures or microbiome-metabolome evidence of impaired regulatory metabolism	Provides a testable adjunctive intervention strategy in stratified clinical studies	Dose, formulation, target population, durability, and clinical endpoints remain insufficiently defined	(113, 153, 166)
Fecal microbiota transplantation	Fecal microbiota transplantation has been proposed as a more direct microbiome-modifying intervention, but evidence in RA remains exploratory	May remodel gut microbial ecology and alter microbial metabolite output, mucosal immune activation, and systemic inflammatory tone	Highly selected refractory RA patients within controlled research settings, particularly when marked dysbiosis or gut-related comorbidity is present	Conceptually relevant for microbiome reprogramming studies	Donor selection, engraftment durability, infection risk, safety, long-term immune effects, and efficacy remain uncertain	(150, 171)
Gut-directed adjunctive stratification	Integration of microbiome, metabolomic, barrier, serological, imaging, and synovial molecular data may help identify biologically defined patient subgroups	May connect mucosal immune disturbance, immune-metabolic remodeling, systemic inflammation, and local synovial pathology	Preclinical or early seropositive RA, MTX-intolerant patients, refractory RA with metabolic syndrome, barrier impairment, or inflammatory bowel disease-like dysbiosis	Most realistic current role is adjunctive stratification, treatment-tolerance prediction, and hypothesis generation	Requires standardized sampling, longitudinal profiling, validated endpoints, and prospective interventional trials	(167, 173, 174)

## 5 Challenges in early stratification driven by immunological heterogeneity and the transition toward precision diagnosis

Although RA diagnosis and management now integrate serological testing, imaging assessment, and targeted treatment strategies, precise early stratification remains challenging (15, 118). RA does not progress through a single linear pathogenic pathway, but represents a heterogeneous disease spectrum shaped by multiple upstream drivers, immune networks, and tissue-level remodeling (119). Current diagnostic tools are effective for identifying established inflammation, autoantibody status, and structural damage, but they are less able to define the dominant immune mechanisms driving disease progression or to predict future structural damage and treatment response (177). Therefore, the diagnostic paradigm is gradually shifting from disease confirmation toward mechanistic stratification and risk prediction (178, 179).

Current clinical stratification mainly relies on RF, ACPAs, erythrocyte sedimentation rate (ESR), C-reactive protein (CRP), and imaging findings (98, 180). These markers remain indispensable for routine diagnosis and standardized clinical assessment, but they largely reflect humoral autoimmunity, systemic inflammatory burden, or established tissue injury rather than upstream pathogenic circuitry (181). This limitation is particularly evident in seronegative patients, preclinical high-risk individuals, and patients with discordant clinical activity, imaging progression, or treatment response (182). Thus, conventional diagnostic models are insufficient for capturing the full spectrum of RA immunological heterogeneity (179, 183).

Recent advances in synovial molecular pathology, multi-omics biomarkers, single-cell profiling, and multimodal data integration have created opportunities to refine RA stratification (184, 185). Molecular information from immune-cell states, cytokine networks, synovial pathotypes, autoantibody profiles, and gut-joint axis-related markers may complement conventional clinical assessment (97, 186). However, these approaches require standardized assays, reproducible classifiers, prospective validation, and demonstration of clinical utility before routine implementation (187, 188). Accordingly, the following sections discuss the limitations of traditional serological markers and the translational value of synovial pathology, multi-omics biomarkers, and multimodal integration in early precision stratification.

### 5.1 Limitations of conventional serological and inflammatory markers in identifying heterogeneity

Conventional serological and inflammatory markers remain central to RA diagnosis and clinical assessment. RF and ACPAs, together with ESR, CRP, and imaging findings, provide standardized information on humoral autoimmunity, systemic inflammatory burden, and structural involvement (98, 180). Among these markers, ACPAs have high diagnostic specificity and are associated with a higher risk of erosive disease, making them particularly useful in early RA identification (98). However, from the perspective of immunological heterogeneity and precision stratification, these markers have limited mechanistic resolution (14, 77).

The first limitation is incomplete sensitivity across disease stages and phenotypes. RF has limited specificity and may be positive in other autoimmune diseases, chronic infections, and elderly individuals (189). Although ACPAs are more specific, a substantial proportion of patients remain seronegative, especially in early, atypical, or less antibody-dominant disease subsets (190, 191). Moreover, RF and ACPAs mainly reflect humoral immune activation and do not indicate whether the dominant pathological process is driven by myeloid inflammation, Th17-related pathways, synovial stromal remodeling, or gut-joint axis-related immune-metabolic changes (111, 186, 192).

The second limitation is their inconsistent ability to predict disease activity, structural progression, and treatment response. High-titer RF/ACPA co-positivity is generally associated with a higher risk of progression, but patients with similar antibody profiles can differ substantially in synovial pathotype, erosion rate, and response to targeted therapy (193). ESR and CRP reflect downstream inflammatory output and are influenced by infection, metabolic status, age, medication exposure, and comorbidities (194). In some patients, systemic inflammatory markers may remain low despite active synovitis or ongoing local tissue damage (195).

A third limitation is that conventional markers provide mainly peripheral and time-point-specific information. They cannot dynamically capture how RA pathogenic networks change across disease stages, tissue compartments, or treatment exposure (196). Therefore, while RF, ACPAs, ESR, and CRP remain indispensable for diagnosis and routine monitoring, they are insufficient as stand-alone tools for mechanistic stratification or individualized treatment prediction (197). Future diagnostic frameworks should retain these markers as baseline clinical anchors while integrating synovial molecular pathology, circulating multi-omics features, microbiome-related information, and multimodal imaging to support pathogenesis-informed stratification (5).

### 5.2 Synovial molecular pathology and synovial pathotype stratification

Compared with peripheral markers, synovial tissue provides more direct information on the cellular composition, activation state, spatial organization, and stromal remodeling of the local pathogenic network in RA (198). With the development of ultrasound-guided minimally invasive synovial biopsy, digital pathology, single-cell profiling, and spatial transcriptomics, synovial molecular pathology has become an important research platform for tissue-level stratification (199). Histological and molecular studies have identified major synovial pathotypes, including lympho-myeloid, diffuse-myeloid, and pauci-immune/fibroid patterns. These pathotypes broadly reflect different dominant programs: lymphoid and humoral immune activation in the lympho-myeloid pathotype, innate myeloid amplification in the diffuse-myeloid pathotype, and stromal activation with matrix remodeling in the pauci-immune/fibroid pathotype (200). Compared with peripheral blood markers, synovial pathology may therefore provide higher mechanistic resolution for assessing local inflammatory circuits, erosion risk, and treatment-response heterogeneity (201).

Among available clinical studies, R4RA and STRAP provide the most direct tests of whether synovial pathobiology can inform targeted therapy selection in RA (202). R4RA moved this concept beyond observational association by embedding synovial biopsy into a randomized treatment framework (64). In anti-TNF-inadequate responders, patients were stratified by synovial B-cell molecular status and randomized to rituximab or tocilizumab. The key finding was that low or absent synovial B-cell lineage signatures identified a group with poor response to B-cell depletion and relatively better response to IL-6 receptor blockade (64). Subsequent molecular analysis further refined this interpretation: humoral immune response modules were associated with rituximab response, whereas B-cell-poor and myeloid-rich profiles were more compatible with tocilizumab responsiveness (64). R4RA also identified stromal/FLS-associated signatures in patients refractory to multiple therapies, suggesting that treatment resistance may sometimes arise from a tissue-embedded, stromal-dominant disease state rather than from failure to suppress a single soluble cytokine (49).

However, STRAP and STRAP-EU complicate a simple biomarker-driven interpretation (202). In bDMARD-naive patients with inadequate response to csDMARDs, synovial biopsy was used to classify patients as B-cell poor or B-cell rich before randomization to rituximab, tocilizumab, or etanercept. Contrary to a straightforward extension of the R4RA hypothesis, the dichotomous B-cell-poor versus B-cell-rich classification did not predict superior or inferior response to rituximab compared with alternative bDMARD strategies at the primary 16-week endpoint (202). This negative result is clinically important because it indicates that synovial B-cell abundance alone is insufficient as a universal treatment-allocation rule (198). Predictive performance may depend on treatment line, prior biologic exposure, endpoint definition, biopsy site, tissue-processing method, and the molecular resolution of the classifier.

Taken together, R4RA and STRAP define both the promise and the current boundary of synovial precision medicine in RA (64). R4RA supports the biological plausibility and clinical relevance of tissue-based molecular subtyping, particularly in treatment-experienced patients in whom the choice between B-cell depletion and IL-6 receptor blockade is clinically meaningful. STRAP cautions that biopsy-driven stratification cannot yet be reduced to a binary B-cell algorithm across all RA populations (198). More recent deep molecular profiling of STRAP synovial biopsies suggests that RNA-seq-based and nCounter-converted models incorporating multiple gene modules, cell-state signatures, and drug-target-related pathways may outperform the original dichotomous B-cell classification (203). Therefore, the next translational step is not simply to increase biopsy use, but to develop reproducible multidimensional synovial classifiers that integrate lymphoid, myeloid, stromal, cytokine, and treatment-resistance programs (165).

**Figure 9:**
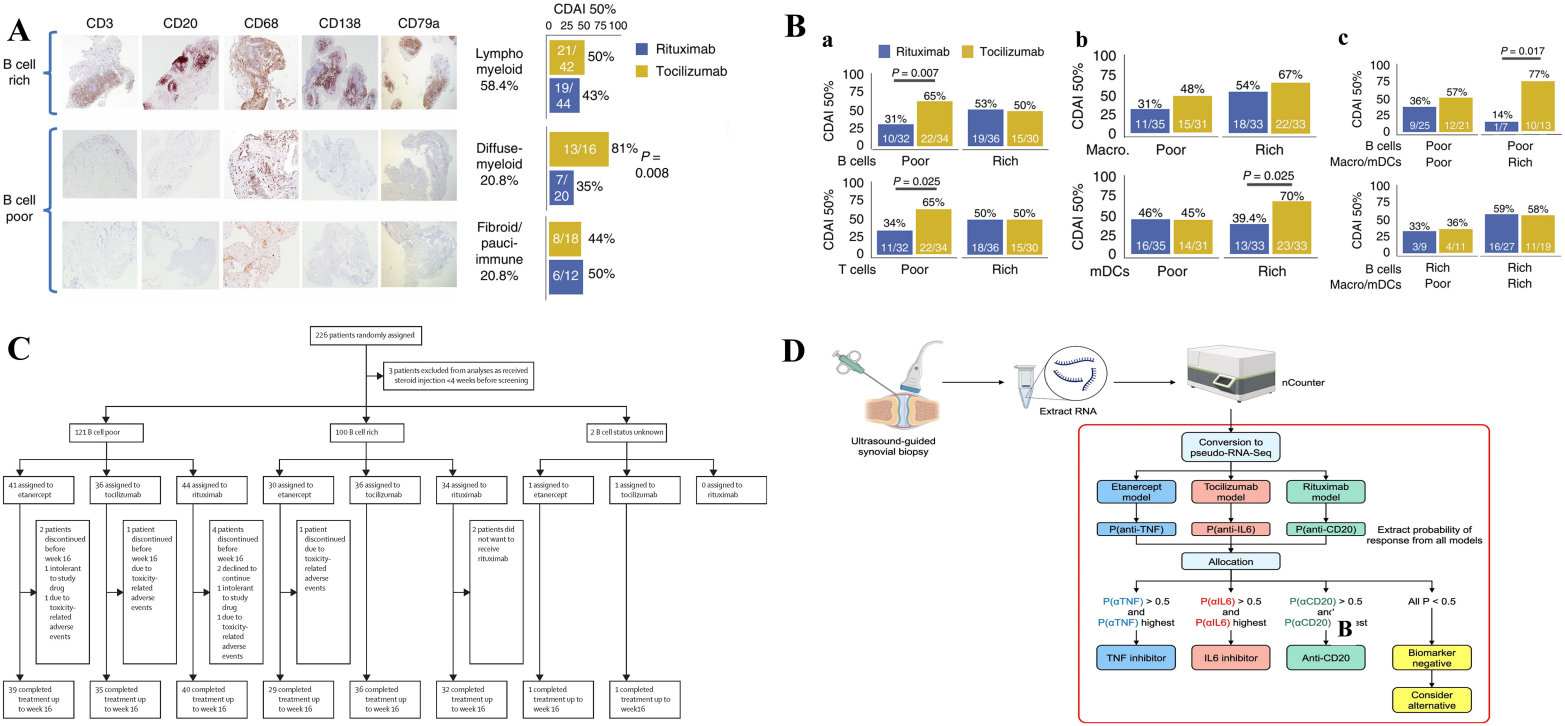
Biopsy-driven synovial stratification and molecular prediction of treatment response in RA. **(A)** Baseline synovial histological pathotypes and their association with 16-week CDAI50 responses to rituximab and tocilizumab in the R4RA trial. **(B)** Treatment responses stratified by synovial B-cell, T-cell, macrophage, and myeloid dendritic cell signatures, showing cell-state-dependent differences in response to rituximab and tocilizumab. **(C)** Trial profile of the STRAP/STRAP-EU biopsy-driven randomized trials in biologic-naïve RA patients stratified by synovial B-cell status. **(D)** Synovial biopsy RNA-based nCounter workflow and molecular classifier-based treatment allocation algorithm for predicting response to TNF inhibition, IL-6 inhibition, or B-cell depletion. Adapted and summarized from refs (64, 202, 203).

Overall, synovial molecular pathology provides a promising foundation for RA precision stratification, but routine clinical implementation remains limited by biopsy standardization, tissue heterogeneity, assay cost, analytic reproducibility, and the need for prospective validation (198, 201) (**Figure 9**). At present, synovial pathotyping should be viewed as a probabilistic and evidence-weighted tool to complement clinical, serological, imaging, and peripheral multi-omics data, rather than as a stand-alone replacement for empirical treatment sequencing (202).

### 5.3 Translational potential of multi-omics biomarkers and circulating immune features

Because conventional markers have limited mechanistic resolution and synovial biopsy is not yet suitable for routine use in all patients, peripheral multi-omics and circulating immune profiling have become important approaches for non-invasive RA stratification. Peripheral blood, serum, plasma, and extracellular vesicle-based samples are accessible, repeatable, and suitable for longitudinal monitoring (132, 133, 204). Multi-omics approaches can capture genomic, transcriptomic, epigenomic, proteomic, metabolomic, and single-cell immune information, thereby complementing conventional clinical, serological, imaging, and synovial pathology data (205).

Different omics platforms provide non-equivalent information (205). Transcriptomic profiling may reflect systemic inflammatory programs and pathway activation; epigenetic features may indicate durable immune-cell regulatory states; proteomic and circulating protein panels may capture effector inflammatory mediators; and metabolomics may reveal immune-metabolic outputs related to glucose, lipid, amino acid, and redox pathways (105, 154). High-dimensional flow cytometry, mass cytometry, and single-cell RNA sequencing can further define circulating immune-cell subsets, including Th17-skewed responses, B-cell activation, and inflammatory monocyte expansion (119). Extracellular vesicles and circulating nucleic acids may provide additional information related to tissue-derived inflammatory signals, although their tissue specificity remains uncertain (204, 206).

From a translational perspective, the main value of multi-omics profiling is not the accumulation of more biomarkers, but the construction of interpretable molecular classifiers (205). Because RA heterogeneity involves multiple biological layers, single omics markers are unlikely to capture the full pathogenic network (207). Integrated models combining transcriptomic, proteomic, metabolomic, single-cell, serological, and imaging data may improve early risk stratification, identification of seronegative or high-risk individuals, and treatment-response prediction (203, 208). However, these models must be evaluated for reproducibility, interpretability, and clinical utility rather than only statistical performance (179, 205).

Not all multi-omics platforms currently have the same level of clinical evidence (205). Transcriptomics, particularly synovial bulk and single-cell RNA-seq, has the strongest evidence base for defining synovial pathotypes and generating treatment-response signatures (203). Peripheral blood transcriptomics may serve as a complementary systemic readout, but it cannot be assumed to fully replace synovial tissue profiling (203, 209). By contrast, proteomics and metabolomics are valuable for capturing downstream effector states, but they remain more sensitive to sample handling, analytical variability, dietary and medication effects, and insufficient cross-cohort validation (210).

A rational biomarker strategy should therefore prioritize clinically interpretable combinations rather than indiscriminate data aggregation. One plausible approach is to integrate baseline transcriptomic signatures reflecting myeloid or lymphoid activation with RF/ACPAs and power Doppler ultrasound assessment of synovial vascularity (203, 211). This combination may jointly capture systemic autoimmunity, local inflammatory anatomy, and molecular drivers of synovial pathology. A key unresolved question is whether peripheral liquid biopsy can approximate synovial transcriptomic information when predicting targeted therapy response (206, 208). For example, circulating extracellular vesicle or exosomal RNA signatures, combined with ultrasound and conventional serology, may help predict primary non-response to TNF inhibitors, but this remains a testable hypothesis rather than a validated clinical rule (44, 212). Prospective biomarker-driven trials are needed before multi-omics integration can become a routine non-invasive triage tool in precision rheumatology (167, 213).

### 5.4 Multimodal integration strategies for early precision stratification

Given the multidimensional nature of RA heterogeneity, multimodal integration should be viewed as a staged clinical triage strategy rather than as exhaustive data aggregation. Its purpose is to connect accessible clinical signals with mechanism-informative biomarkers, thereby identifying patients who may require deeper molecular assessment and more refined treatment-response prediction (214, 215). This approach is particularly relevant for seronegative disease, atypical or undifferentiated arthritis, early treatment failure, and refractory RA, where conventional markers may not fully capture the dominant pathogenic program (49, 183, 216).

Artificial intelligence and machine learning may further support this staged integration by linking heterogeneous clinical and molecular data into probabilistic stratification models. Recent studies suggest that machine learning approaches can integrate clinical variables, serology, imaging-derived inflammatory features, peripheral transcriptomic signatures, synovial RNA profiles, and multi-omics biomarkers to identify latent patient subgroups and predict treatment response. In biopsy-driven RA cohorts, synovial RNA-seq-based machine learning models have shown predictive value for response to etanercept, tocilizumab, and rituximab, and their conversion into nCounter-based assays illustrates a potential route toward clinically deployable molecular classifiers (203). Whole-blood transcriptomic models may provide a less invasive complementary strategy for predicting response to TNF inhibition, although their relationship to synovial tissue states remains incomplete (217). More broadly, AI-assisted integration may help move RA stratification from isolated biomarkers toward probabilistic, mechanism-informed patient grouping (218, 219). However, these models remain limited by small training cohorts, platform heterogeneity, overfitting, insufficient external validation, limited interpretability, and uncertain cost-effectiveness. Therefore, AI/ML-based subtyping should currently be viewed as an investigational decision-support approach rather than a replacement for clinical assessment, serology, imaging, or biopsy-driven validation.

A clinically feasible integration pathway should begin with standardized first-line assessment. Conventional serology, inflammatory markers, and imaging remain the foundation because they are accessible, reproducible, and embedded in routine clinical workflows (220, 221). When these tools provide concordant information, additional high-dimensional testing may not be necessary. However, when clinical phenotype, serological status, imaging activity, and treatment response are discordant, escalation to synovial molecular pathology or selected circulating molecular markers may provide added mechanistic information (201, 222).

In this stepwise framework, synovial tissue profiling may help determine whether local disease is predominantly lymphoid, myeloid, or stromal, while circulating biomarkers may provide complementary information on systemic immune activation, drug exposure, inflammatory burden, or immune-metabolic status (203, 214). The value of integration therefore lies not in combining all available platforms, but in selecting the minimum set of clinically interpretable markers according to specific clinical questions, thereby supporting diagnosis refinement, progression-risk assessment, primary non-response prediction, and reassessment after treatment failure (205, 213).

Despite its translational promise, multimodal integration faces important methodological and implementation challenges (208). These include inconsistent sample collection, variability in pre-analytical processing, non-standardized computational pipelines, limited cross-cohort reproducibility, and the limited interpretability of some machine-learning models (208, 223). Future studies should prioritize transparent algorithms, standardized workflows, clinically meaningful endpoints, and prospective trials that test whether integrated models improve decision-making beyond existing clinical, serological, and imaging frameworks. In this context, multimodal integration represents a promising direction for RA precision stratification, but its clinical value will depend on whether it can generate reproducible, interpretable, and actionable predictions (179, 205).

### 5.5 Knowledge gaps and testable hypotheses for future research

Although multi-omics integration and precision stratification have expanded the conceptual framework of RA diagnosis, several knowledge gaps continue to limit mechanism-guided clinical implementation (224). A central limitation is that most biomarker studies remain associative: molecular signatures are often linked to disease phenotypes, retrospective outcomes, or treatment responses, but few have been tested prospectively as tools for selecting therapy (225). Future research should therefore move from descriptive molecular profiling toward biomarker-driven trials that compare molecularly guided strategies with standard treat-to-target care (202). Three testable hypotheses are particularly relevant.

First, it remains unclear whether peripheral liquid biopsy can approximate the mechanistic resolution of synovial tissue profiling (177). A clinically testable hypothesis is that circulating extracellular vesicle or exosomal RNA signatures, when integrated with baseline power Doppler ultrasound and conventional serology, may predict primary non-response to TNF inhibitors with performance comparable to synovial biopsy-based transcriptomic models (44, 212). Validation of this hypothesis would support the development of non-invasive and longitudinal molecular triage strategies, but would not eliminate the need for synovial tissue profiling in selected patients (177, 226).

Second, the temporal role of the gut-joint axis during targeted therapy remains insufficiently defined (144, 186). Current studies often describe microbial dysbiosis as an upstream disease-associated feature, but its contribution to longitudinal treatment response and difficult-to-treat RA is less clear (227). A second hypothesis is that baseline depletion of selected SCFA-producing taxa may contribute to secondary loss of response to IL-6 receptor antagonists by weakening immunometabolic regulation of Th17/Treg balance, intestinal barrier integrity, and systemic inflammatory tone (113, 228). This concept could be tested in longitudinal trials that combine biologic therapy with standardized microbiome or metabolite-directed interventions (165). If supported, these studies would suggest that some forms of secondary treatment failure may involve immunometabolic remodeling rather than purely pharmacological mechanisms (105, 164).

Third, the relationship between synovial pathotypes and localized intervention remains underexplored (201). Current treatment strategies for difficult-to-treat RA still largely rely on systemic escalation or cycling of bDMARDs and tsDMARDs, yet some patients remain inadequately controlled. Spatial and single-cell studies suggest that pauci-immune/fibroid synovial pathotype may be associated with treatment resistance through stromal remodeling and fibrogenic FLS programs rather than immune-cell-dominant inflammation. A third hypothesis is that patients with a confirmed pauci-immune/fibroid synovial pathotype may benefit from localized, microenvironment-responsive delivery systems designed to modulate FLS and the synovial niche, compared with repeated systemic biologic cycling (229, 230). This remains an investigational concept, and validation would require pathotype-defined patient selection, disease-relevant delivery platforms, and controlled comparison with standard systemic strategies (231, 232).

Together, these hypotheses emphasize that the next stage of RA precision medicine should not simply generate larger biomarker catalogs. Instead, future studies should test whether specific molecular signatures can improve clinical decisions, identify biologically defined patient subgroups, and predict response to targeted interventions in prospective and reproducible settings (**Table 4**).

**Table 4:** Stratification tools for early precision assessment in RA: evidence strength, clinical utility, and limitations.

Stratification tool	Sample or data source	Information captured	Main clinical use	Evidence strength	Key limitation	References
RF	Serum	Humoral autoimmunity and autoantibody-associated disease risk	Routine diagnostic support and baseline serological classification	Routine clinical use	Limited specificity; may be positive in other autoimmune diseases, chronic infections, and elderly individuals	(233, 234)
ACPAs	Serum	Citrullinated antigen-directed humoral autoimmunity, seropositive disease status, and erosion-risk tendency	Routine diagnosis, prognosis, and identification of ACPA-positive RA	Routine clinical use	Incomplete sensitivity; seronegative RA remains common, and ACPA positivity alone does not define the dominant pathogenic mechanism	(235, 236)
RF/ACPA co-positivity and antibody burden	Serum	Stronger humoral autoimmunity, autoantibody breadth, and higher-risk serological profile	Risk assessment for structural progression and more aggressive disease course	Routine clinical use with prognostic value	Patients with similar antibody profiles may still differ in synovial pathotype, erosion rate, and therapeutic response	(237, 238)
ESR and CRP	Peripheral blood	Downstream systemic inflammatory burden and acute-phase response	Routine monitoring of disease activity and treatment response	Routine clinical use	Influenced by infection, age, metabolic status, medication exposure, and comorbidities; may not reflect active local synovitis in some patients	(239, 240)
Conventional radiography	Joint imaging	Established structural damage, erosions, and joint-space narrowing	Baseline structural assessment and monitoring of irreversible damage	Routine clinical use	Limited sensitivity for early inflammatory activity and pre-erosive synovial pathology	(239, 241)
Ultrasound	Joint imaging	Synovial hypertrophy, effusion, tenosynovitis, and local inflammatory activity	Early detection of synovitis and assessment of clinically ambiguous disease activity	Established adjunctive clinical tool	Operator dependence and inter-center variability; molecular mechanisms cannot be directly inferred	(242, 243)
Power Doppler ultrasound	Joint imaging	Synovial vascularity and active inflammatory perfusion	Assessment of active synovitis, progression risk, and treatment-response monitoring	Established adjunctive clinical tool	Signal variability depends on equipment, acquisition settings, operator expertise, and inflammatory context	(244, 245)
MRI	Joint imaging	Synovitis, bone marrow edema, tenosynovitis, erosions, and early structural involvement	Sensitive assessment of early inflammatory and structural lesions	Established adjunctive tool in selected settings	Cost, accessibility, standardization, and limited direct molecular specificity restrict routine use for all patients	(246, 247)
Synovial biopsy	Synovial tissue	Local cellular composition, immune-cell infiltration, stromal remodeling, and tissue-level inflammatory organization	Research-based tissue stratification and mechanistic assessment of local disease programs	Promising but not routine	Invasiveness, biopsy-site heterogeneity, tissue-processing variability, cost, and limited routine availability	(64, 248)
Synovial molecular pathology	Synovial tissue; histology, transcriptomics, single-cell profiling, spatial analysis	Local immune and stromal programs, synovial pathotypes, cytokine activity, and treatment-resistance signatures	Mechanism-informed tissue stratification and refinement of treatment-response prediction	Promising but not routine	Requires standardized biopsy workflows, reproducible classifiers, and prospective validation before routine implementation	(64, 249)
R4RA biopsy-driven evidence	Synovial biopsy linked to randomized treatment allocation	Synovial B-cell lineage status, humoral immune modules, myeloid-rich profiles, and stromal/FLS-associated resistance signatures	Evidence that synovial molecular features may inform biologic selection in treatment-experienced RA	Strong translational proof-of-concept	Findings are context-dependent and mainly applicable to selected treatment-experienced populations	(64, 80)
STRAP and STRAP-EU biopsy-driven evidence	Synovial biopsy linked to randomized treatment allocation	B-cell-poor versus B-cell-rich classification and response to rituximab, tocilizumab, or etanercept	Tests whether synovial B-cell status can guide biologic selection in bDMARD-naive patients	Important negative or boundary-setting evidence	Binary B-cell classification was insufficient as a universal treatment-allocation rule; more multidimensional classifiers are needed	(202, 203)
Transcriptomics	Peripheral blood or synovial tissue	Inflammatory pathway activity, myeloid or lymphoid activation signatures, interferon-related programs, and treatment-response modules	Molecular stratification and treatment-response prediction	Strongest omics evidence, especially for synovial profiling	Peripheral blood transcriptomics cannot be assumed to fully replace synovial tissue profiling; cross-cohort reproducibility remains essential	(250, 251)
Proteomics and circulating protein panels	Serum, plasma, synovial fluid, or tissue-derived samples	Effector inflammatory mediators, cytokine patterns, tissue-remodeling proteins, and downstream disease activity signals	Candidate biomarkers for disease activity, progression risk, and therapeutic response	Promising but not routine	Sensitive to sample handling, analytical platform, inflammation status, and cross-cohort variability	(252, 253)
Metabolomics	Serum, plasma, synovial fluid, stool-related metabolic profiles	Immune-metabolic outputs related to glucose, lipid, amino acid, redox, and microbial metabolite pathways	Immune-metabolic profiling and adjunctive risk or treatment-response assessment	Investigational	Strongly influenced by diet, medication exposure, sample type, analytical platform, and disease activity	(210, 254)
Single-cell RNA-seq and high-dimensional cytometry	Synovial tissue or peripheral blood immune cells	Immune-cell states, macrophage subsets, T-cell and B-cell programs, stromal-cell activation, and cellular heterogeneity	High-resolution mechanistic stratification and discovery of cell-state signatures	Investigational with strong mechanistic value	Cost, technical complexity, data interpretation, batch effects, and limited routine feasibility	(255, 256)
Extracellular vesicle or exosomal RNA	Peripheral blood, serum, plasma, or extracellular vesicle-enriched samples	Circulating RNA signals potentially reflecting systemic or tissue-derived inflammatory activity	Non-invasive molecular triage and longitudinal monitoring	Testable hypothesis/requires validation	Tissue specificity remains uncertain, and predictive performance must be tested against synovial biopsy-based models	(257, 258)
Multimodal integration	Combined clinical phenotype, serology, imaging, synovial pathology, circulating biomarkers, and treatment-response data	Concordance or discordance among clinical activity, serological status, imaging inflammation, tissue pathology, and molecular biomarkers	Stepwise clinical triage, diagnosis refinement, progression-risk assessment, primary non-response prediction, and reassessment after treatment failure	Promising but not routine	Requires transparent algorithms, standardized workflows, interpretable models, clinically meaningful endpoints, and prospective validation	(208, 259)
Biomarker-driven clinical trial design	Prospective molecularly guided treatment allocation	Whether molecular signatures improve decision-making beyond standard treat-to-target care	Validation of mechanism-informed treatment selection and precision stratification	Required for clinical implementation	Few biomarkers have been tested prospectively as tools for selecting therapy	(64, 203)
Peripheral liquid biopsy plus imaging hypothesis	Extracellular vesicle or exosomal RNA, power Doppler ultrasound, and conventional serology	Non-invasive approximation of synovial inflammatory or transcriptomic states	Potential prediction of primary non-response to TNF inhibitors	Testable hypothesis	Requires prospective comparison with synovial biopsy-based transcriptomic models	(260, 261)
Gut-joint axis-related stratification hypothesis	Microbiome, metabolomics, barrier-related markers, and clinical treatment outcomes	Microbial and immune-metabolic features potentially linked to treatment response or secondary loss of response	Adjunctive stratification in selected patients, especially difficult-to-treat or metabolically defined RA	Investigational	Causality, patient selection, standardized sampling, and intervention endpoints remain insufficiently defined	(186, 262)
Synovial pathotype-guided local intervention hypothesis	Synovial biopsy, spatial profiling, single-cell analysis, FLS-associated signatures, and delivery-platform assessment	Pauci-immune/fibroid synovial pathotype, stromal remodeling, and tissue-embedded treatment resistance	Selection of patients for localized or microenvironment-responsive delivery approaches in research settings	Investigational	Requires pathotype-defined patient selection, disease-relevant delivery platforms, and controlled comparison with systemic strategies	(177, 263)

## 6 Immune heterogeneity-related non-response to targeted therapy and its mechanistic basis

Targeted therapies have substantially improved RA management by enabling selective inhibition of key cytokine pathways, immune-cell interactions, and intracellular signaling cascades (264). However, despite the availability of bDMARDs and tsDMARDs, primary non-response, secondary loss of response, flare after dose tapering or discontinuation, and failure to achieve sustained remission remain common clinical challenges (51). These outcomes cannot be explained solely by drug potency or treatment timing (197). Instead, they are closely related to the immunological heterogeneity of RA, including differences in dominant immune-cell programs, cytokine dependence, synovial pathotypes, tissue remodeling, and gut-joint axis-related systemic features (102, 265).

Clinically, non-response does not necessarily indicate that a drug has no biological activity (19, 43). In many cases, it may reflect a mismatch between the therapeutic target and the dominant pathogenic pathway in a given patient at a given disease stage (4, 266). Alternatively, initially effective therapy may lose efficacy because the disease network evolves under treatment pressure (267, 268). Potential mechanisms include compensatory activation of bypass signaling pathways, shifts in effector-cell states, stromal or fibrotic remodeling of the synovial microenvironment, altered drug exposure, and the development of ADAs (96, 199). Therefore, understanding treatment non-response requires moving beyond single-target matching toward an integrated framework that considers disease stage, tissue context, immune-network redundancy, and longitudinal remodeling (254).

Accordingly, this section focuses on three related aspects of treatment-response heterogeneity: the variable clinical benefit of current targeted therapies across different immunological and synovial pathotypes, the contribution of target-pathway mismatch to primary non-response, and the emergence of secondary loss of response during prolonged treatment exposure (203). Clarifying these aspects may help explain why patients with the same clinical diagnosis respond differently to targeted therapies and may support more evidence-based strategies for treatment selection, switching, and combination approaches in RA (269).

### 6.1 Current targeted therapy strategies and heterogeneous clinical benefit

Current targeted therapies for RA include bDMARDs targeting TNF-α, IL-6 receptor signaling, T-cell co-stimulation, or B cells, and tsDMARDs represented mainly by JAK inhibitors (270, 271). These agents have substantially improved disease control, but clinical benefit remains heterogeneous across patients (4, 272). This variability indicates that treatment response is not determined solely by drug class, but also by whether the targeted pathway is biologically relevant in the patient’s current disease state (227).

In clinical practice, TNF inhibitors, IL-6 receptor antagonists, T-cell co-stimulation modulators, B-cell depletion therapy, and JAK inhibitors are often selected according to disease activity, comorbidities, safety profile, prior treatment exposure, and physician experience (273). However, conventional clinical and serological measures cannot reliably determine whether a patient’s disease is primarily driven by myeloid inflammation, adaptive immune activation, stromal remodeling, or mixed pathway dependence (4). Therefore, apparent differences in drug efficacy should be interpreted as a consequence of underlying immune heterogeneity rather than as simple superiority or inferiority of one drug class over another (274).

This section therefore uses current targeted therapies as a clinical entry point to discuss treatment non-response (117). The key question is not only which drug is available, but whether the selected therapeutic target matches the dominant inflammatory program at a given disease stage (272). This provides the basis for distinguishing primary non-response from secondary loss of response and for developing more rational treatment-switching strategies (275).

### 6.2 The immunological basis of primary non-response

Primary non-response refers to the failure to achieve an adequate clinical response after initiation of an appropriately selected targeted therapy (191). In RA, this phenomenon often reflects a mismatch between the selected therapeutic target and the dominant pathogenic mechanism active in a given patient, rather than simply inadequate dosage, poor adherence, or insufficient drug potency (266). Thus, primary non-response should be interpreted as a clinical manifestation of underlying immune heterogeneity (226, 276).

One major mechanism is variability in pathway dependence (114, 277). RA inflammation may be driven predominantly by TNF-α/NF-κB signaling, IL-6/JAK/STAT3 signaling, Th17-associated pathways, B-cell-mediated humoral immunity, or stromal programs centered on FLS activation (184). If the selected agent blocks a pathway that is not central to the patient’s current disease state, clinical improvement may be limited even when the drug successfully inhibits its intended target (3). This issue is particularly relevant in patients with established synovial remodeling, in whom FLS activation, extracellular matrix remodeling, angiogenesis, and fibrosis may reduce dependence on a single soluble cytokine and promote a more tissue-embedded inflammatory state (278, 279).

Adaptive immune organization and network redundancy may further contribute to primary non-response (30). In some patients, persistent T-cell help, B-cell activation, and TLS formation may sustain local autoreactive immune responses (280). In others, several inflammatory pathways may be active at baseline, making single-target blockade insufficient to reach a clinically meaningful anti-inflammatory threshold (13, 281). Host genetic background, epigenetic regulation, post-transcriptional programs, mucosal immune status, metabolic remodeling, and gut-joint axis-related features may also influence immune-cell sensitivity to targeted intervention (101, 143). These factors suggest that primary non-response is rarely explained by one mechanism alone (227).

From a translational perspective, primary non-response highlights the limitations of empirical treatment selection (203). Pre-treatment assessment integrating synovial pathotypes, circulating immune signatures, cytokine dependence, serological status, imaging findings, and selected gut-joint axis-related markers may improve prediction of which patients are unlikely to respond to a given targeted therapy (46, 102). However, such models require prospective validation before they can be used for routine treatment allocation (282).

Overall, primary non-response in RA reflects the interaction of pathway mismatch, immune-network redundancy, tissue-level remodeling, and persistent upstream pathogenic drivers (283). Targeted therapy therefore does not automatically equal precision therapy (284, 285). Its success depends on whether the selected mechanism of action is sufficiently aligned with the patient’s dominant disease program at the time of treatment initiation (27, 57). Clarifying these mechanisms may support pre-treatment stratification and more rational individualized therapy selection (6, 286).

### 6.3 Secondary loss of response and mechanisms of therapeutic escape

Secondary loss of response refers to the recurrence of disease activity, reduced inflammatory control, or structural progression after an initial clinical response to targeted therapy (287). Unlike primary non-response, it reflects a dynamic process in which the disease network changes under prolonged therapeutic pressure (4, 43). Potential mechanisms include compensatory activation of alternative signaling pathways, synovial microenvironment remodeling, altered pharmacokinetics, ADAs, persistent adaptive immune activation, and metabolic adaptation (229).

Compensatory signaling is a major mechanism of therapeutic escape (111, 288). Because cytokine and intracellular signaling networks in RA are redundant, chronic blockade of one pathway may allow alternative inflammatory circuits to sustain disease activity (192). For example, suppression of TNF-α- or JAK/STAT-related signaling may be followed by increased activity of NF-κB-, MAPK-, interferon-, IL-17-, or other cytokine-associated programs in selected patients (124). This does not imply that the initial therapy was biologically ineffective; rather, it indicates that inflammatory networks can reorganize over time and reduce dependence on the originally targeted pathway (289).

Synovial tissue remodeling may also contribute to secondary loss of response (51, 112). During chronic disease, FLS activation, extracellular matrix deposition, angiogenesis, fibrosis, and altered matrix-adhesion signaling may promote a more tissue-embedded inflammatory state (123, 264). In this context, local synovial pathology may persist even when systemic inflammatory markers improve (123, 290). Persistent tissue-level inflammation and stromal activation can therefore maintain cartilage and bone damage despite partial systemic control (31, 154).

Adaptive immune persistence and pharmacokinetic factors represent additional mechanisms (94, 291). In some patients, autoreactive B cells, long-lived plasma cells, and TLS may continue to support local antigen presentation and autoantibody production, allowing inflammatory amplification to re-emerge after an initial response (85). For bDMARDs, ADAs may reduce drug exposure by accelerating clearance or neutralizing drug activity, thereby contributing to loss of efficacy in susceptible patients (12). Cellular phenotypic switching and metabolic remodeling in macrophages, FLS, and endothelial cells may further support persistence of the synovial pathogenic niche (31, 185). Gut-joint axis-related factors, including dysbiosis, barrier dysfunction, and Th17/Treg imbalance, may also contribute to systemic inflammatory persistence, although their role in secondary loss of response requires further validation (102, 143).

Overall, secondary loss of response reflects the evolution of RA immune heterogeneity under treatment pressure (112). It may arise from bypass signaling, synovial niche consolidation, persistent adaptive immunity, ADAs, and metabolic adaptation (222). These mechanisms provide the rationale for longitudinal monitoring and treatment reassessment, which are discussed in the following section (142).

### 6.4 Optimizing personalized therapy through molecular subtyping and dynamic monitoring

The persistence of primary non-response and secondary loss of response indicates that RA treatment selection still relies partly on empirical sequencing rather than mechanism-informed matching (51). Although the therapeutic arsenal has expanded, the key challenge is to identify which immune or tissue-level program is dominant in a given patient at a given disease stage (272). Molecular subtyping may help align clinical phenotype, synovial pathotype, therapeutic target, and timing of intervention, but its use must remain evidence-weighted and clinically feasible (112, 290).

A practical personalized strategy should begin with defining the dominant pathogenic program (126, 292). Patients may differ in the relative contribution of myeloid inflammation, adaptive immune activation, Th17 polarization, B-cell-driven humoral immunity, FLS-mediated stromal remodeling, and gut-joint axis-related systemic features (102, 160). Integrating clinical phenotype, serological status, imaging findings, synovial pathology, and selected molecular biomarkers may help classify patients into more interpretable disease patterns, such as lympho-myeloid, diffuse-myeloid, or pauci-immune/fibroid synovial pathotypes (97, 209). However, these categories should be viewed as probabilistic disease states rather than fixed labels.

For patients with inadequate response, reassessment should focus on identifying the mechanism of treatment failure rather than simply repeating empirical drug cycling (27). In primary non-response, the key issue is whether the selected therapeutic target was mismatched with the dominant disease program at baseline (57). In secondary loss of response, the priority is to distinguish reduced drug exposure or immunogenicity from evolving pathway dependence, synovial remodeling, or broader immune-metabolic adaptation (101). This distinction can be supported by integrating disease activity measures, imaging progression, drug concentrations, ADAs, and selected molecular or tissue-level biomarkers (124, 150).

Because RA immune heterogeneity changes over time, personalized therapy should not rely only on baseline classification (177, 289). Longitudinal monitoring may help detect pathway switching, emerging stromal-dominant persistence, pharmacokinetic failure, or recurrent inflammatory activity before irreversible structural progression occurs (264, 272). Predictive models should therefore prioritize interpretability, reproducibility, and clinical usability rather than unstructured accumulation of high-dimensional data (26, 191).

At present, molecular subtyping-guided RA management remains in a translational phase (64, 292). Most predictive models still require validation in large, multicenter, prospective cohorts, and high-resolution molecular diagnostics are unlikely to be widely implemented in routine practice in the near term (182, 203). Overall, molecular subtyping and dynamic monitoring may help move RA management from empirical sequencing toward more rational, mechanism-informed therapy selection, but their clinical value will depend on prospective validation and demonstrable improvement over current treat-to-target strategies (41, 289).

## 7 From inflammation control toward immune rebalancing: emerging intervention strategies and translational prospects

Current targeted therapies have substantially improved inflammation control and clinical outcomes in RA (117). However, many patients still experience incomplete remission, primary non-response, secondary loss of response, or relapse after dose reduction or discontinuation (51, 269). This indicates that blocking a single cytokine, immune-cell interaction, or intracellular signaling pathway may not be sufficient to durably reshape the broader pathogenic network in all patients. RA is sustained by interacting layers of immune heterogeneity, synovial microenvironment remodeling, mucosal immune disturbance, and systemic metabolic reprogramming (27, 229). Therefore, future therapeutic development should not focus only on stronger downstream inflammation suppression, but also on strategies that modulate upstream immune drivers, tissue-level persistence, and mechanisms related to immune regulation (9, 94).

In this context, emerging interventions should be viewed as complementary and investigational strategies rather than immediate replacements for established targeted therapies (54, 270). Some approaches aim to reduce upstream inflammatory inputs through microbiome modulation, metabolic intervention, or mucosal barrier support (38). Others focus on the local synovial microenvironment by using targeted delivery systems or microenvironment-responsive platforms to improve tissue specificity and reduce systemic exposure (293, 294). Additional strategies, including MSC-based therapies and cell-engineering approaches, attempt to modulate immune regulation or autoreactive immune-cell compartments, but their durability, safety, and clinical positioning in RA remain to be defined (101). Overall, the field is gradually moving from inflammation suppression alone toward immune-network rebalancing and tissue-contextualized intervention (31). Whether these approaches can improve sustained remission or reduce treatment escape will require rigorous preclinical validation, controlled clinical trials, and careful patient selection based on molecular and tissue-level disease features (267).

### 7.1 Microbiome and immunometabolic interventions as adjunctive strategies

Based on the potential involvement of the gut-joint axis in RA heterogeneity, microbiome and immunometabolic interventions have attracted interest as adjunctive strategies targeting upstream inflammatory inputs (102). Unlike conventional targeted therapies that mainly inhibit downstream cytokines or signaling pathways, these approaches aim to modulate microbial composition, mucosal barrier function, gut-derived metabolites, and systemic metabolic-inflammatory states (79, 264). For example, SCFA-related pathways may influence mucosal immune regulation and Th17/Treg balance, providing a mechanistic rationale for gut- and metabolism-directed interventions in selected patients (228, 295). However, their ability to modify RA progression or prevent relapse remains insufficiently validated in prospective clinical studies (106).

Current approaches include probiotics, prebiotics, synbiotics, dietary modification, and metabolite-oriented strategies (38, 296). Their potential value should not be interpreted simply as increasing “beneficial bacteria,” but as reshaping functional microbial outputs, including SCFA production, tryptophan metabolism, bile acid signaling, lactate accumulation, and lipid metabolic balance (43). These pathways may influence mucosal immune tone, epithelial barrier integrity, systemic inflammation, and possibly synovial immune activation, although their relative contribution is likely to vary across patient subsets (174).

Clinically, microbiome and immunometabolic interventions should currently be positioned as complementary strategies rather than substitutes for established DMARD-based therapy (174, 297). They may be most relevant for patients with evidence of gut-joint axis disturbance, persistent low-grade inflammation, metabolic comorbidity, or relapse tendency after treatment tapering (141). Major barriers include inter-individual microbiome variability, dietary and geographic confounding, medication effects, lack of standardized intervention protocols, and limited long-term efficacy data (26, 54). Therefore, future studies should focus on patient selection, standardized microbiome-metabolome endpoints, and controlled trials that test whether these interventions can add measurable benefit to existing RA treatment strategies (210, 298).

### 7.2 Targeted delivery systems and local synovial microenvironment modulation

Targeted delivery systems provide a potential strategy for improving drug exposure within inflamed joints while limiting systemic toxicity (298, 299). Unlike microbiome or metabolic interventions that mainly target upstream systemic inputs, local delivery approaches focus on the synovial microenvironment, where inflammatory cells, activated FLS, angiogenesis, hypoxia, ROS accumulation, acidic pH, and increased MMPs activity create disease-associated delivery cues (57). These features may be exploited to enhance tissue retention, trigger local drug release, or improve the spatiotemporal specificity of antirheumatic therapy (3, 300). However, their translational relevance should be interpreted cautiously because RA is usually systemic and polyarticular, and uniform delivery to multiple small joints remains difficult (27, 33).

Current delivery strategies mainly include biomimetic targeting, ligand-mediated targeting, and microenvironment-responsive release (229, 293). Biomimetic nanoparticles coated with macrophage-, platelet-, erythrocyte-, or synovial cell-derived membranes may reduce immune clearance and improve inflammation-directed accumulation in experimental models (79). Ligand-modified systems can further enhance interaction with activated macrophages or FLS through receptors such as folate receptor β, CD44, integrins, or scavenger receptors (57, 301). In parallel, pH-, ROS-, or MMP-responsive polymers and hydrogels can be designed to release therapeutic payloads preferentially within inflamed synovial tissue (302). These approaches may improve local drug availability, but their long-term safety, reproducibility, and clinical superiority over conventional local formulations remain to be established (296).

Multi-payload co-delivery platforms represent another investigational direction (303). Because RA inflammatory networks are redundant, coordinated delivery of anti-inflammatory drugs, kinase-modulating agents, or nucleic acid therapeutics may theoretically target multiple pathogenic layers simultaneously (264). For example, combining small-molecule agents with siRNA or regulatory microRNAs could modulate both upstream inflammatory mediators and downstream stromal or osteoclast-related pathways (54, 264). Nevertheless, such strategies remain largely preclinical, and their complexity raises challenges related to dosing, release kinetics, off-target effects, manufacturing consistency, and regulatory evaluation (153).

From a translational perspective, localized reformulation of approved antirheumatic agents using relatively mature platforms, such as liposomes, hydrogels, microspheres, or albumin-associated systems, currently appears more clinically feasible than highly complex systemic smart nanomedicines (304, 305). These approaches may be particularly relevant for refractory monoarticular or oligoarticular inflammation, where prolonged intra-articular retention and reduced systemic exposure are clinically meaningful (54). By contrast, systemically administered multi-component nanocarriers face major barriers, including heterogeneous joint distribution, limited accumulation across polyarticular disease sites, batch-to-batch variability, quality-control difficulties, protein corona formation, complement activation, immunogenicity, long-term biodistribution, and cumulative toxicity (13, 93).

Overall, targeted delivery systems may improve the precision of local intervention in selected RA settings, but they should currently be viewed as adjunctive and investigational rather than established disease-modifying strategies (226, 293). Near-term translation is more likely to come from pragmatic local formulation optimization using clinically tractable materials, whereas highly complex smart nanosystems remain better suited for mechanistic exploration and carefully designed preclinical-to-clinical validation (208, 306).

### 7.3 Immunomodulatory potential of MSC-based therapy

MSC-based therapy has attracted interest as an investigational immunomodulatory strategy for RA, particularly in refractory disease settings where single-pathway blockade is insufficient (27). Unlike conventional biologics that target defined cytokines or immune-cell interactions, MSCs may influence multiple immune and tissue-repair pathways through paracrine signaling, cell-cell communication, and modulation of the inflammatory microenvironment (143). However, their role in RA should be interpreted as a potential immune-regulatory approach rather than as an established method for immune rebalancing (43, 177).

Mechanistically, MSCs may modulate both innate and adaptive immune responses (46, 281). Experimental and early clinical studies suggest that MSCs can reduce macrophage and DC activation, decrease production of inflammatory mediators such as TNF-α and IL-6, suppress Th1 and Th17 polarization, and support Treg function (84, 210). MSC-derived secretomes may also exert tissue-protective effects by regulating oxidative stress, angiogenesis, extracellular matrix degradation, and osteoclast-related pathways within the synovial microenvironment (96, 265). These effects provide a biological rationale for MSC-based therapy in RA, but the magnitude and durability of benefit remain variable across studies (54, 64).

From a clinical translation perspective, MSC-based therapy should not be regarded as a first-line or broadly established alternative to DMARD-based treatment (208, 284). Its more realistic positioning is as an investigational strategy for selected refractory patients, especially those with complex immune-network activation or inadequate responses to csDMARDs, bDMARDs, and tsDMARDs (46, 307). Major barriers include heterogeneity in tissue source, donor variability, expansion protocols, dosing, route of administration, *in vivo* persistence, homing efficiency, and manufacturing quality control (54, 154). In addition, long-term safety, efficacy durability, and optimal patient selection remain insufficiently defined (165, 266).

Cell-free derivatives, including MSC-derived extracellular vesicles and exosomes, may offer a more standardized and scalable direction for future development (188, 204). These products could potentially retain part of the immunomodulatory activity of parent cells while reducing some challenges related to live-cell manufacturing and persistence (82, 308, 309). Nevertheless, they also require rigorous characterization, potency assays, biodistribution studies, and controlled clinical validation (41, 113). Overall, MSC-based approaches remain promising but investigational, and their future role in RA will depend on standardized manufacturing, mechanistic patient selection, and high-quality clinical evidence.

### 7.4 CAR-T and other engineered cell-based therapies in RA

CAR-T therapy and other engineered cell-based approaches represent highly experimental strategies in RA (310). Unlike conventional targeted therapies that inhibit soluble cytokines, immune-cell interactions, or intracellular signaling pathways, engineered cellular therapies are designed to modify or eliminate selected immune-cell populations that may contribute to persistent autoimmunity (13, 311, 312). Their theoretical rationale is based on the observation that disease persistence and relapse may involve autoreactive B cells, pathogenic T-cell subsets, plasma-cell niches, and local immune memory within synovial tissue (57, 209). However, this rationale should be distinguished from established clinical efficacy in RA (292).

CAR-NK cells represent another emerging engineered-cell platform with theoretical advantages over conventional CAR-T cells, including off-the-shelf allogeneic manufacturing, lower risk of graft-versus-host disease, and potentially reduced CRS and ICANS. Recent autoimmune-disease studies have begun to explore this concept. An iPSC-derived CD19/BCMA dual-targeting CAR-NK product was reported in a patient with severe systemic sclerosis, and allogeneic CD19 CAR-NK therapy has also been evaluated in relapsed or refractory systemic lupus erythematosus (313, 314). These early findings suggest that CAR-NK platforms may offer a scalable and potentially safer route for B-cell or plasma-cell-directed immune depletion in selected autoimmune diseases. However, RA-specific CAR-NK clinical evidence remains absent or extremely limited, and current support is extrapolated mainly from oncology, systemic lupus erythematosus, and systemic sclerosis studies. Major unresolved issues include short *in vivo* persistence, optimal target selection, tissue trafficking into inflamed synovium, durability of immune reset, repeated dosing requirements, manufacturing standardization, and long-term safety. Therefore, CAR-NK therapy should be considered a distant investigational direction for refractory RA rather than a near-term therapeutic option.

Recent reports of CD19-directed CAR-T therapy in refractory systemic autoimmune diseases have stimulated interest in applying this approach to RA (315). In principle, B-cell-directed CAR-T strategies could reduce autoreactive B-cell activity and modify humoral immune networks in selected refractory patients (280). Other engineered platforms, including regulatory T-cell-based approaches or synthetic suppressor-cell systems, have also been proposed to enhance antigen-specific or tissue-restricted immune regulation (316). Nevertheless, RA-specific evidence remains limited, and most concepts are still at an early translational stage (27, 182).

From a clinical translation perspective, CAR-T therapy currently has low priority for routine RA management (317). Several issues limit broad application, including uncertain target selection, patient-selection ambiguity, safety concerns, manufacturing complexity, high cost, and the need for specialized treatment infrastructure (299). Unlike hematological malignancies, RA lacks a clearly disease-specific cellular antigen comparable to a tumor lineage antigen (3, 318). Potential targets may overlap with physiological immune compartments, raising concerns about excessive immune depletion, off-target effects, or incomplete removal of pathogenic clones (46, 100). In addition, CAR-T therapy can be associated with cytokine release syndrome, immune effector cell-associated neurotoxicity syndrome, cytopenia, hypogammaglobulinemia, infection risk, and complications related to lymphodepleting preconditioning (319, 320). Although autoimmune-disease studies may show milder toxicity profiles than oncology settings, these risks require careful justification in a chronic non-malignant disease (321).

Therefore, CAR-T and related engineered cell therapies should currently be considered investigational options rather than established RA treatments (322). Their potential role may be limited to highly selected patients with severe refractory disease, marked humoral autoimmunity, repeated failure of conventional targeted therapies, or major extra-articular involvement, preferably within controlled clinical-trial settings (272, 310). Future development will require improved antigen specificity, controllable safety switches, transient or *in vivo* CAR expression systems, reduced toxicity, standardized monitoring, and long-term follow-up (280). Until these issues are resolved, engineered cell therapies in RA should be viewed as a frontier research direction rather than a near-term routine therapeutic strategy (210).

### 7.5 Practical bottlenecks and clinical translation challenges in the immune rebalancing strategies

Microbiome modulation, immunometabolic intervention, targeted delivery systems, MSC-based therapy, and engineered cell therapies provide new directions for RA treatment beyond conventional inflammation suppression (293, 323). However, these approaches remain difficult to translate into scalable clinical strategies (208). RA is not driven by a single pathogenic mechanism, but by dynamic interactions among genetic susceptibility, mucosal immunity, systemic inflammatory networks, synovial microenvironment remodeling, and treatment-induced immune adaptation (77, 177). Therefore, tolerance-oriented or immune-rebalancing strategies require more than a single technological breakthrough; they require accurate patient selection, mechanism-based matching, durable efficacy, safety, standardization, and clinical accessibility (267).

The first major challenge is patient selection (57). Not all patients require intensive immune remodeling, and not all refractory patients share the same mechanism of treatment failure (298). Emerging strategies may be most relevant for patients with multi-pathway activation, stromal-dominant synovial persistence, mucosal immune disturbance, or organized humoral immune activity (193). Without clear mechanistic stratification, there is a risk of overtreating patients who may respond to standard therapy while missing those who might benefit from advanced interventions (18). In addition, conventional endpoints such as disease activity scores, inflammatory markers, and radiographic progression may not fully capture the effects of immune-regulatory or microenvironment-directed strategies (105, 192). Future trials may need composite endpoints that include sustained remission, relapse after tapering, drug-sparing outcomes, imaging evidence of synovial persistence, and selected molecular or cellular markers (324).

The second challenge is durability and safety (267, 325). Because RA pathogenic networks can compensate and remodel over time, initial improvement after an immune-regulatory intervention may not translate into sustained benefit (191, 281). Disease activity may recur through bypass signaling, synovial niche re-consolidation, persistent mucosal immune abnormalities, or residual immune memory (294). At the same time, deeper immune modulation introduces additional safety concerns (298). Microbiome and metabolic interventions may have broad systemic effects; targeted delivery systems require evaluation of carrier distribution, accumulation, degradation, and long-term toxicity; and MSC- or CAR-T-based approaches require careful assessment of infection, abnormal immune responses, immune depletion, and long-term stability (291). Because RA is a chronic non-malignant disease, any advanced strategy must demonstrate a favorable long-term risk-benefit profile before broad clinical adoption (292).

The third challenge is standardization, manufacturability, and integration with existing care (27, 57). Microbiome interventions lack unified functional targets, metabolic interventions may depend heavily on individual baseline profiles, targeted delivery systems face complex formulation and quality-control requirements, and cell-based therapies are limited by manufacturing variability, high cost, regulatory complexity, and specialized infrastructure (173, 323). Their clinical positioning also remains uncertain: whether they should be used as early adjunctive interventions, rescue strategies for refractory disease, maintenance approaches after remission, or tools to support drug tapering has not been clearly defined (85).

Overall, tolerance-oriented strategies represent an important future direction in RA, but their translation depends on mechanistic subtyping, dynamic monitoring, precision trial design, and rational integration with existing DMARD-based treatment (282, 326). Rather than replacing established therapies, these approaches are more likely to develop as adjunctive or highly selected interventions (296, 305). Their clinical value will depend on whether they can produce reproducible, safe, and measurable benefits beyond current treat-to-target strategies (41, 326).

### 7.6 Mechanism-oriented integrated strategies for precision RA management

To connect molecular stratification with therapeutic optimization, this review summarizes an integrated reference framework for mechanism-informed RA management (327). This framework provides a structured way to consider how clinical phenotype, serological status, synovial pathotype, circulating immune features, treatment response, drug exposure, and emerging therapeutic options may be combined to support future precision stratification and therapeutic optimization (46, 112).

At baseline, molecular and tissue-level information may help estimate which pathogenic programs are most active in an individual patient (112). A lympho-myeloid synovial pathotype, particularly when accompanied by high RF/ACPAs burden, CXCL13-related signatures, or Tfh/B-cell-associated transcriptomic features, may suggest stronger involvement of humoral and lymphoid immune programs (78). In selected contexts, this profile could support consideration of B-cell depletion or T-cell co-stimulation blockade, although treatment prediction cannot rely on these markers alone (78). By contrast, diffuse-myeloid synovial pathotype with myeloid activation, IL-6-related transcriptional programs, or elevated systemic inflammatory markers may indicate greater relevance of TNF-α, IL-6, or JAK/STAT-targeted strategies (328, 329). Pauci-immune/fibroid synovial pathotype, characterized by relatively low leukocyte infiltration and stronger stromal or FLS-associated signatures, may be associated with reduced sensitivity to conventional cytokine-directed therapy and may justify further evaluation of JAK inhibition, stromal-targeted strategies, or localized delivery approaches in research settings (18, 96).

When treatment failure occurs, the framework first separates primary non-response from secondary loss of response (51, 112). In primary non-response, reassessment should focus on target-pathway mismatch and on whether the current clinical phenotype still reflects active inflammation, stromal persistence, or non-inflammatory pain (326). Where feasible, imaging, serological reassessment, synovial pathology, or blood-based molecular profiling may help determine whether a different pathogenic program is dominant (330). If persistent inflammatory activity is confirmed, mechanism-informed treatment adjustment may be more rational than automatic same-class cycling, while still requiring integration with safety, comorbidities, patient preference, and existing clinical guidelines (27, 290).

For secondary loss of response, the framework emphasizes distinguishing pharmacokinetic failure from pharmacodynamic escape (51, 269). In patients treated with bDMARDs, therapeutic drug monitoring, including drug trough levels and ADAs, may help identify reduced exposure or immunogenicity-related loss of efficacy (274). If ADAs and low drug concentrations are present, switching within the same mechanistic class or optimizing concomitant MTX may be reasonable in selected patients (22, 331). Conversely, if drug concentrations are adequate but disease activity recurs, bypass signaling, pathway switching, or synovial microenvironment remodeling should be considered (272). In this setting, repeat clinical assessment, imaging, and molecular re-profiling may support mechanism-informed treatment adjustment rather than simple drug cycling (112).

Advanced interventions, including MSC-based therapy and CAR-T or other engineered cell therapies, should be placed at the outer edge of this framework (280). These approaches are not established routine RA treatments and should be considered only as investigational options for highly selected refractory patients, preferably within controlled clinical-trial settings (113). CD19-directed CAR-T therapy may be conceptually relevant for severe humoral autoimmunity or refractory B-cell-driven disease, whereas MSC-based or MSC-derived extracellular vesicle strategies may be more relevant to broader immune-regulatory or stromal-remodeling contexts (332). Their use requires careful assessment of long-term safety, infection risk, immune depletion, manufacturing feasibility, cost, and patient selection (298).

**Figure 10:**
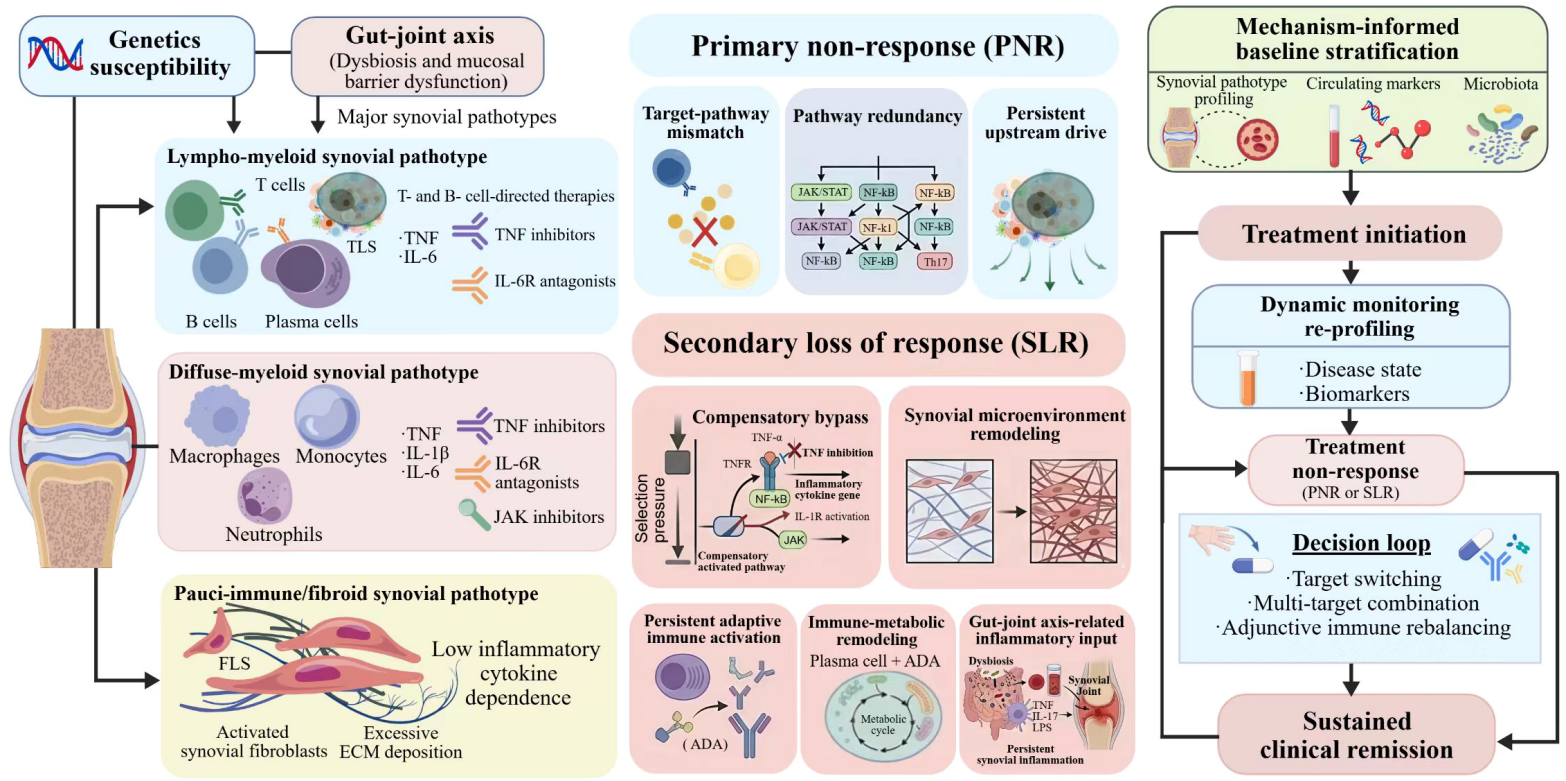
Mechanism-informed stratification and dynamic management of treatment non-response in RA. Genetic susceptibility, gut-joint axis-related immune-metabolic interactions, and synovial pathotypes may contribute to dominant pathogenic programs and treatment-response heterogeneity in RA. Primary non-response may reflect target–pathway mismatch, cytokine-network redundancy, or persistent upstream inflammatory drivers, whereas secondary loss of response may involve compensatory pathway activation, synovial microenvironment remodeling, persistent adaptive immune activation, immune-metabolic remodeling, anti-drug antibodies, and gut-joint axis-related inflammatory input. A practical management framework should combine mechanism-informed baseline stratification, treatment initiation, dynamic monitoring, treatment-response reassessment, and mechanism-informed therapeutic adjustment to reduce empirical treatment cycling and support sustained clinical remission.

Overall, this integrated reference framework organizes RA precision management around four interconnected steps: mechanism-informed baseline stratification, reassessment after primary non-response, dynamic monitoring during secondary loss of response, and cautious evaluation of advanced investigational strategies in selected refractory disease (26, 101). By linking molecular heterogeneity with clinical decision points, this framework may help reduce empirical treatment cycling and support more rational therapeutic optimization in future prospective studies (210) (**Figure 10**).

## 8 Conclusions and future perspectives

RA should be understood as a heterogeneous and dynamically evolving autoimmune disease in which genetic susceptibility (70), epigenetic and post-transcriptional regulation, autoantibody diversification, mucosal immune disturbance, gut microbiome-related immune-metabolic changes, cytokine-network redundancy, and synovial microenvironment remodeling jointly shape disease progression and treatment response (14). This heterogeneity indicates that clinically similar patients may differ substantially in dominant immune programs, synovial pathotypes, structural progression risk, and therapeutic sensitivity (177). Therefore, RA management should move beyond static disease classification toward immune-subtype-informed clinical decision-making.

In this framework, conventional treat-to-target care remains the foundation, with RF, ACPAs, ESR, CRP, and imaging serving as indispensable first-line tools (333). When clinical phenotype, serological status, imaging activity, and treatment response are concordant, routine management may be sufficient (334). However, in seronegative, atypical, high-risk, refractory, or early treatment-failure subsets, deeper assessment using synovial molecular pathology, circulating multi-omics biomarkers, drug exposure monitoring, and selected microbiome or metabolomic indicators may help refine immune subtyping (97, 327). Such stratification should aim to identify whether disease activity is predominantly humoral/lymphoid, myeloid/cytokine-driven, stromal/tissue-embedded, immune-metabolic, or pharmacokinetically influenced, rather than to assign patients to a rigid one-marker-one-drug category (81, 177).

A practical immune-subtype-informed strategy should link baseline mechanism assessment with longitudinal reassessment (335). Primary non-response should prompt reconsideration of target-pathway mismatch and dominant pathogenic programs, whereas secondary loss of response should be evaluated for ADAs, reduced drug exposure, compensatory signaling, pathway switching, or persistent synovial remodeling (279, 287). Emerging approaches, including microbiome modulation, immunometabolic intervention, targeted delivery, MSC-based therapy, CAR-T/CAR-NK or other engineered cell therapies, should currently be positioned as adjunctive or investigational options for selected patients rather than established immune-rebalancing therapies (336). Future progress will depend on reproducible molecular classifiers, externally validated AI/ML-assisted models, prospective biomarker-guided trials, standardized monitoring workflows, and clinically feasible algorithms that reduce empirical treatment cycling while improving prediction, safety, and durable remission (337).
