# Supplementary material for: Immunological heterogeneity in rheumatoid arthritis: challenges in early-stage stratification, non-response to targeted therapy, and the restoration of immune tolerance
Source: Front Immunol. 2026 Jul 20;17:1894263. doi: 10.3389/fimmu.2026.1894263 (PMC13429679; doi:10.3389/fimmu.2026.1894263)
Supplement: Supplementary file 1 [file DataSheet1.pdf]

Supplementary Table 1. Glossary of key concepts and standardized terminology used in this review.

Key concept	Standardized term used in this review	Definition
Rheumatoid arthritis	RA	A chronic systemic autoimmune disease.
Immunological heterogeneity	Immunological heterogeneity	Differences in immune mechanisms among RA patients and across different disease stages.
Molecular subtyping	Molecular subtyping	Classification of RA patients based on molecular-, cellular-, or tissue-level features.
Synovial molecular pathology	Synovial molecular pathology	Analysis of synovial tissue using histology, transcriptomics, single-cell analysis, or spatial omics to identify local pathogenic immune programs and stromal remodeling features.
Synovial pathotype	Synovial pathotype	A tissue-level inflammatory and remodeling pattern defined by dominant immune cells, stromal components, and molecular features in synovial tissue.
Lympho-myeloid synovial pathotype	Lympho-myeloid synovial pathotype	A synovial pathotype characterized by prominent lymphoid and myeloid cell infiltration.
Diffuse-myeloid synovial pathotype	Diffuse-myeloid synovial pathotype	A synovial pathotype characterized by dominant myeloid-cell activation and inflammatory macrophage-related programs.
Pauci-immune/fibroid synovial pathotype	Pauci-immune/fibroid synovial pathotype	A synovial pathotype characterized by relatively low leukocyte infiltration and more prominent stromal remodeling, fibrosis, or FLS-associated programs.
Autoantibody diversification	Autoantibody diversification	Expansion and qualitative variation in the breadth and features of autoreactive antibody responses.
Gut-joint axis	Gut-joint axis	A potential cross-organ regulatory network linking gut microbiome alterations, mucosal immunity, microbial metabolites, systemic inflammation, and joint pathology.
Immune-metabolic profiles	Immune-metabolic profiles	Patterns of immune and metabolic activity shaped by microbial metabolites, systemic inflammation, tissue metabolic states, and treatment exposure.
Primary non-response	Primary non-response	Failure to achieve an adequate clinical response after appropriately selected targeted therapy.
Secondary loss of response	Secondary loss of response	Recurrence of disease activity or reduced therapeutic efficacy after an initial treatment response.
Mechanism-informed therapy selection	Mechanism-informed therapy selection	Treatment selection based on the dominant immune, synovial, molecular, or pharmacological mechanisms in an individual patient.
Multimodal integration	Multimodal integration	Stepwise integration of clinical phenotype, serology, imaging, synovial pathology, circulating biomarkers, and longitudinal treatment-response information to support precision stratification in RA.
Multi-omics biomarkers	Multi-omics biomarkers	Biomarkers derived from genomics, epigenomics, transcriptomics, proteomics, metabolomics, single-cell analysis, or extracellular vesicle-based assays.
Immune rebalancing strategies	Immune rebalancing strategies	Interventions or therapeutic strategies aimed at promoting a more regulated immune state.
Tolerance-oriented strategies	Tolerance-oriented strategies	Strategies designed to enhance immune regulation or reduce pathological immune activation.
