# Supplementary material for: Immunological heterogeneity in rheumatoid arthritis: challenges in early-stage stratification, non-response to targeted therapy, and the restoration of immune tolerance
Source: Front Immunol. 2026 Jul 20;17:1894263. doi: 10.3389/fimmu.2026.1894263 (PMC13429679; doi:10.3389/fimmu.2026.1894263)
Supplement: Supplementary file 2 [file DataSheet2.pdf]

Supplementary Table 2. Abbreviations used in this review.

Abbreviation	Full term
RA	Rheumatoid arthritis
ACPA/ACPAs	Anti-citrullinated protein antibody/antibodies
FLS	Fibroblast-like synoviocytes
Th17	T helper 17 cells
IL-6	Interleukin-6
TNF/TNF-α	Tumor necrosis factor / tumor necrosis factor-α
MSC/MSCs	Mesenchymal stem/stromal cell(s)
Treg/Tregs	Regulatory T cell(s)
RF	Rheumatoid factor
CAR-T	Chimeric antigen receptor T-cell therapy
MMP/MMPs	Matrix metalloproteinase(s)
HLA	Human leukocyte antigen
P. copri	Prevotella copri
ADA/ADAs	Anti-drug antibody/antibodies
IL-17	Interleukin-17
JAK/STAT	Janus kinase/signal transducer and activator of transcription pathway
SCFA/SCFAs	Short-chain fatty acid(s)
ncRNA/ncRNAs	Non-coding RNA(s)
AMPA/AMPAs	Anti-modified protein antibody/antibodies
DC/DCs	Dendritic cell(s)
NF-κB	Nuclear factor kappa B
R4RA	R4RA biopsy-driven RA clinical trial
STRAP/STRAP-EU	STRAP and STRAP-EU synovial biopsy-driven RA clinical studies
bDMARDs	Biological disease-modifying antirheumatic drugs
m6A	N6-methyladenosine
Tfh	Follicular helper T cells
CRP	C-reactive protein
DMARD/DMARDs	Disease-modifying antirheumatic drug(s)
ESR	Erythrocyte sedimentation rate
STAT3	Signal transducer and activator of transcription 3
tsDMARDs	Targeted synthetic disease-modifying antirheumatic drugs
IC/ICs	Immune complex(es)
IL-1β	Interleukin-1β
IL-12	Interleukin-12
IL-23	Interleukin-23
JAK/STAT3	Janus kinase/signal transducer and activator of transcription 3 pathway
MAPK	Mitogen-activated protein kinase
MMP-1/MMP-3/MMP-13	Matrix metalloproteinase-1/-3/-13
MTX	Methotrexate
ROS	Reactive oxygen species
SE	Shared epitope

Abbreviation	Full term
SOCS/SOCS3	Suppressor of cytokine signaling / suppressor of cytokine signaling 3
TLS	Tertiary lymphoid structures
Th1	T helper 1 cells
anti-CCP	Anti-cyclic citrullinated peptide antibody/antibodies
csDMARDs	Conventional synthetic disease-modifying antirheumatic drugs
AhR	Aryl hydrocarbon receptor
CD19	Cluster of differentiation 19
CD4+	Cluster of differentiation 4-positive cells
CXCL/CXCL13	C-X-C motif chemokine ligand / C-X-C motif chemokine ligand 13
DAMP/DAMPs	Damage-associated molecular pattern(s)
HLA-DRB1	Human leukocyte antigen-DRB1
IL-10	Interleukin-10
MERTK	MER proto-oncogene tyrosine kinase
NET/NETs/NETosis	Neutrophil extracellular trap(s) / neutrophil extracellular trap formation
OPG	Osteoprotegerin
PTPN22	Protein tyrosine phosphatase non-receptor type 22
RANKL	Receptor activator of nuclear factor-κB ligand
RNA-seq	RNA sequencing
TGF-β	Transforming growth factor-β
circRNA/circRNAs	Circular RNA(s)
lncRNA/lncRNAs	Long non-coding RNA(s)
miRNA/miRNAs	MicroRNA(s)
AAPAs	Anti-acetylated protein antibodies
CD206	Cluster of differentiation 206
CD28	Cluster of differentiation 28
CD44	Cluster of differentiation 44
CTLA4	Cytotoxic T-lymphocyte-associated protein 4
ERK	Extracellular signal-regulated kinase
FOXP3	Forkhead box P3
H3K18la	Histone H3 lysine 18 lactylation
H3K9la	Histone H3 lysine 9 lactylation
HBEGF	Heparin-binding EGF-like growth factor
HLA-DR	Human leukocyte antigen-DR
IL-21	Interleukin-21
JNK	c-Jun N-terminal kinase
RANKL/OPG	RANKL/osteoprotegerin axis or ratio
SLAMF7	Signaling lymphocytic activation molecule family member 7
SPP1	Secreted phosphoprotein 1
STAT4	Signal transducer and activator of transcription 4
TLR	Toll-like receptor
TRAF3IP2	TRAF3-interacting protein 2
ceRNA	Competing endogenous RNA
